# Analysis of auxin responses in the fern *Ceratopteris richardii* identifies the developmental phase as a major determinant for response properties

**DOI:** 10.1242/dev.203026

**Published:** 2024-09-26

**Authors:** Sjoerd Woudenberg, Melissa Dipp Alvarez, Juriaan Rienstra, Victor Levitsky, Victoria Mironova, Enrico Scarpella, Andre Kuhn, Dolf Weijers

**Affiliations:** ^1^ Laboratory of Biochemistry, Wageningen University, Stippeneng 4, 6708WE Wageningen, The Netherlands; ^2^ Institute of Cytology and Genetics, Lavrentyeva Avenue 10, Novosibirsk 630090, Russian Federation; ^3^ Department of Plant Systems Physiology, Radboud University, Heyendaalseweg 135, 6525 AJ Nijmegen, The Netherlands; ^4^ Department of Biological Sciences, University of Alberta, CW-405 Biological Sciences Building, Edmonton AB T6G 2E9, Canada

**Keywords:** Auxin, *Ceratopteris*, Evolution, Plant development, Fern, *Marchantia*, Evo-devo

## Abstract

The auxin signaling molecule regulates a range of plant growth and developmental processes. The core transcriptional machinery responsible for auxin-mediated responses is conserved across all land plants. Genetic, physiological and molecular exploration in bryophyte and angiosperm model species have shown both qualitative and quantitative differences in auxin responses. Given the highly divergent ontogeny of the dominant gametophyte (bryophytes) and sporophyte (angiosperms) generations, however, it is unclear whether such differences derive from distinct phylogeny or ontogeny. Here, we address this question by comparing a range of physiological, developmental and molecular responses to auxin in both generations of the model fern *Ceratopteris richardii*. We find that auxin response in Ceratopteris gametophytes closely resembles that of a thalloid bryophyte, whereas the sporophyte mimics auxin response in flowering plants. This resemblance manifests both at the phenotypic and transcriptional levels. Furthermore, we show that disrupting auxin transport can lead to ectopic sporophyte induction on the gametophyte, suggesting a role for auxin in the alternation of generations. Our study thus identifies developmental phase, rather than phylogeny, as a major determinant of auxin response properties in land plants.

## Teaser

**Summary:** A study of auxin responses in the fern *Ceratopteris* reveal that developmental phase tissue ontogeny, rather than species phylogeny, is a major driver for divergence in the auxin response in land plants.

## INTRODUCTION

Throughout evolution, plants adopted hormone signaling pathways to control their development and their responses to external stimuli. Many of these phytohormones are broadly distributed, and responses are in many cases conserved among all land plant taxa, with components of land plant pathways found even in algal sisters (reviewed by Blázquez et al., 2020; Bowman et al., 2019; Depuydt and Hardtke, 2011; Menand et al., 2007; Wang et al., 2015). Although the initial description of such phytohormone responses and pathways was restricted to angiosperms, notably *Arabidopsis thaliana* (hereafter *Arabidopsis*) (e.g. auxin; Evans et al., 1994; Sieburth, 1999), recent years have seen increased exploration in bryophytes, such as *Physcomitrium patens* (hereafter *Physcomitrium*) (Sakakibara et al., 2003; Thelander et al., 2018) and *Marchantia polymorpha* (hereafter *Marchantia*) (Eklund et al., 2015; Flores-Sandoval et al., 2015; Kato et al., 2015). Responses to phytohormones have thus been recorded in a variety of land plants, but these can hardly be compared because of the divergent morphologies between the different land plant clades. It is therefore difficult to define a clear evolutionary scenario for the hormonal control of plant growth and development. This difficulty is most prominent between bryophytes and tracheophytes, as their dominant generations do not share direct tissue homologies (Harrison, 2017).

Auxin, one of the most-studied and best-understood plant hormones, is crucial for many processes in bryophytes and tracheophytes (reviewed by Kato et al., 2018). The pathway mediating transcriptional auxin responses is conserved among all land plants and partly present in streptophyte algae (Carrillo-Carrasco et al., 2023; Mutte et al., 2018). Auxin controls many different developmental processes, including rhizoid formation in the bryophytes *Marchantia* and *Physcomitrium* (Flores-Sandoval et al., 2015; Sakakibara et al., 2003; Thelander et al., 2018), and root branching at the expense of root elongation in *Arabidopsis* (Casimiro et al., 2001; Dubrovsky et al., 2008; Lavenus et al., 2013). However, because of the lack of direct tissue homology, it is hard to conclude whether those processes are comparable. One exception might exist on a cellular level, as both *Arabidopsis* root hairs and *Marchantia* rhizoids – specified by similar genetic pathways (Jones and Dolan, 2012; Menand et al., 2007) – extend and initiate upon auxin treatment (Flores-Sandoval et al., 2015). Other processes are superficially similar but are not comparable because of the completely different morphology, for example when comparing gravitropism in single-celled rhizoids with multicellular roots (Zhang et al., 2019). Many auxin responses in bryophytes cannot therefore be directly compared with those in tracheophytes, and therefore the question remains as to which part of the response is dependent on the developmental phase (gametophyte/sporophyte) and which is dependent on species phylogeny.

Ferns, as a sister clade to flowering plants, are positioned phylogenetically intermediate between the model bryophytes *Marchantia* and *Physcomitrium*, and the model tracheophyte *Arabidopsis* (Donoghue et al., 2021; Puttick et al., 2018)*.* Most importantly, fern lifestyles closely resemble both (thalloid) bryophytes and tracheophytes (Conway and Di Stilio, 2020). During land plant evolution, a major transition took place from a dominant haploid gametophyte generation in bryophytes to a dominant diploid sporophyte in tracheophytes. In (most) ferns, both these generations are autotrophic and free living. From a haploid spore, a gametophytic prothallus forms that develops rhizoids, archegonia and antheridia, whereas from the diploid embryo, a sporophytic plant develops with leaves and root-hair carrying roots, both innervated by vasculature. There are sparse descriptions of auxin responses in the model fern *Ceratopteris richardii* (hereafter *Ceratopteris*), but no clear overview of responses in both generations has been reported and considered in the context the of the evolution of auxin responses. Auxin treatments on *Ceratopteris* gametophytes are known to affect sexual differentiation, spore germination, rhizoid development, and the positioning and development of the lateral notch meristem (Chauhan, 2024; Gregorich and Fisher, 2006; Hickok and Kiriluk, 1984; Withers et al., 2023b). In gametophytes of other fern species, reported effects also include cell expansion and elongation (Miller, 1961; Miller and Miller, 1965), and antheridium differentiation (Ohishi et al., 2021). Sporophytic roots from *Ceratopteris* show reduced growth rates upon auxin treatments and an increase in adventitious rooting, whereas no effects on lateral root initiation has been reported (Hou et al., 2004; Yu et al., 2020). In sporophytes of other fern species, cell wall extensibility and cell elongation was increased upon auxin treatment in the rachis (Cookson and Osborne, 1979), auxin from the youngest pinnae (‘top’) promote fiddlehead uncoiling, growth and differentiation (Briggs and Steeves, 1959; Peterson and Cutter, 1969; Steeves and Briggs, 1960; Voeller, 1960; Wetmore and Pratt, 1949; White, 1971) whereas – in contrast to flowering plants – auxin was reported not to promote vascular differentiation (Ma and Steeves, 1992).

Here, we have mapped auxin responses in both generations of the fern *Ceratopteris* to uncouple developmental phase from species phylogeny as determinants of auxin responses and to better understand its ancestral role. We find that, phenotypically, *Ceratopteris* gametophytes respond similarly to *Marchantia* gametophytes, whereas *Ceratopteris* sporophytes closely resemble responses of *Arabidopsis* sporophytes. This separation in response is further shown in their profiles of auxin-dependent gene regulation but not in their direct genetic targets. We identify that differences in auxin-dependent gene activation are likely caused by increased levels of Aux/IAA repressor proteins in the sporophyte. Last, we find that auxin can disrupt the alternation of generations. Together, our data identify tissue ontogeny, rather than species phylogeny, as a major driver for divergence in the auxin response in land plants.

## RESULTS

### A thalloid liverwort-like response in the *Ceratopteris* gametophyte

**Fig. 1.**
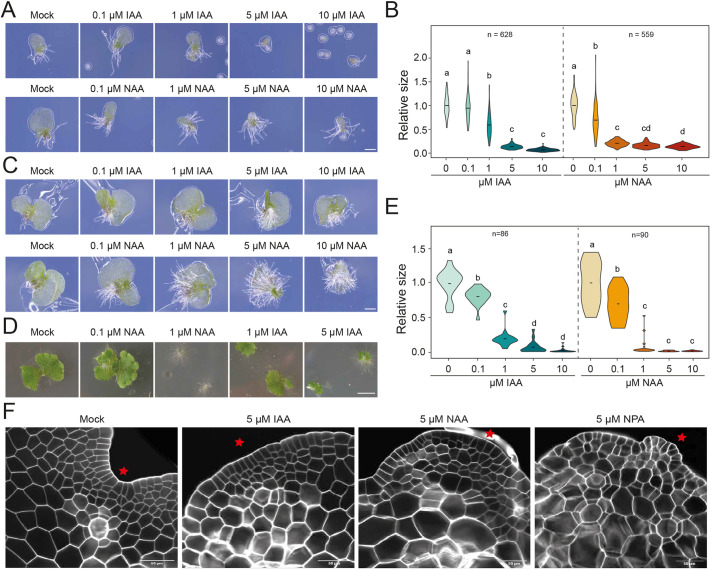
**Auxin response in *Ceratopteris* gametophytes.** (A) *Ceratopteris* gametophytes developed from spores germinated and grown on auxin-supplemented medium (at the indicated concentrations of IAA or NAA) until sexual maturity. (B) Violin plots of the relative *Ceratopteris* thallus size (normalized to mock treatment) upon IAA treatment (left) or NAA treatment (right) of four pooled replicate experiments (*n*>100 *Ceratopteris* gametophytes per treatment). (C) *Ceratopteris* gametophytes 4 days after transfer to auxin-supplemented medium after germination on unsupplemented media (in which they develop a lateral meristem). (D) Twelve-day-old *Marchantia* polymorpha gemmalings grown on medium supplemented with the indicated concentrations of NAA or IAA. (E) Violin plots of quantifications of *Marchantia* gemmae grown of auxin supplemented medium for 12 days. (F) Cellular organization of lateral notch meristems in thallus derived from spores transferred to auxin-supplemented medium after 4 days of germination on hormone-free medium. Red stars indicate the location of the lateral meristem. Scale bars: 0.25 mm in A; 0.5 mm in C; 50 mm in D; 50 µm in F. Statistical significance in B, C and F is shown by letters based on ANOVA and Tukey's pair-wise comparison (*P*<0.05).

To explore the growth responses to externally applied auxin in the *Ceratopteris* gametophyte, spores were placed on media containing different concentrations of the natural auxin indole 3-acetic acid (IAA) or the synthetic auxin 1-naphthyl acetic acid (NAA). Previous research only focused on the effects of exogenous auxin on gametophyte cell numbers and lateral meristem formation (Gregorich and Fisher, 2006; Hickok and Kiriluk, 1984; Withers et al., 2023b). Hermaphrodite gametophytes grown before sexual maturation showed a clear reduction in growth in response to both auxins at all concentrations tested, except at 100 nM IAA, which appeared to slightly promote prothallus growth (Fig. 1A,B). These results are consistent with previously reported concentrations for affecting gametophyte cell numbers upon auxin treatments (Withers et al., 2023b). Under standard conditions, rhizoids only develop close to the spore coat (Conway and Di Stilio, 2020). With NAA, however, they developed ectopically on the thallus border (Fig. S1A). Given the strong growth-inhibiting effect of both auxins on the thallus, it is difficult to disentangle potentially independent effects on growth and rhizoid formation. We therefore transferred sexually immature gametophytes to auxin-supplemented media following initial growth on control media. Upon transfer, a small growth reduction was visible but also a clear induction of (ectopic) rhizoids (Fig. 1C; Fig. S1). This response closely resembles the phenotype in *Marchantia* in qualitative and quantitative terms, although the starting plant material for these experiments is highly different – with spores rather than gemmae (Fig. 1D,E). This suggests that *Marchantia* and *Ceratopteris* sensitivity and reaction to auxin perturbations in long-term growth experiments is similar. Comparisons were made to *Marchantia* due their shared thallus growth habit, in contrast to more leafy bryophytes such as *Physcomitrum*.

Detailed microscopic analysis revealed that the morphology of the *Ceratopteris* prothallus also changed in auxin-treated gametophytes, manifesting as disorganization of the meristematic notch (Fig. 1F; Fig. S1A). Most importantly, not only did we observe these morphological defects in gametophytes treated with external auxin, but also when inhibiting IAA transport with 1-N-naphtylphtalamic acid (NPA; Fig. 1F; Fig. S1A). The connection of auxin action with the development of the lateral meristem is in agreement with previously reported data where intermediate auxin levels specify the placement of the lateral meristem (Withers et al., 2023b), and is also important for notch development in *Marchantia* (Flores-Sandoval et al., 2015). However, we were unable to phenocopy the distorted morphology in *Marchantia* apical notches, which first widen and then disappear upon NPA or NAA treatment (Fig. S2). The product of the *Ceratopteris* lateral meristems is the female sexual organ, which establishes the first major 3D axis and will form the future sporophyte upon fertilization of its egg cell (Conway and Di Stilio, 2020). We did not observe obvious defects in the morphology of the archegonia, even upon NPA treatment (Fig. S1B). Therefore, *Ceratopteris* gametophytes show auxin responses that are similar to those observed in gametophytes of the thalloid liverwort *Marchantia*.

### The *Ceratopteris* sporophyte shows a flowering plant-like auxin response

**Fig. 2.**
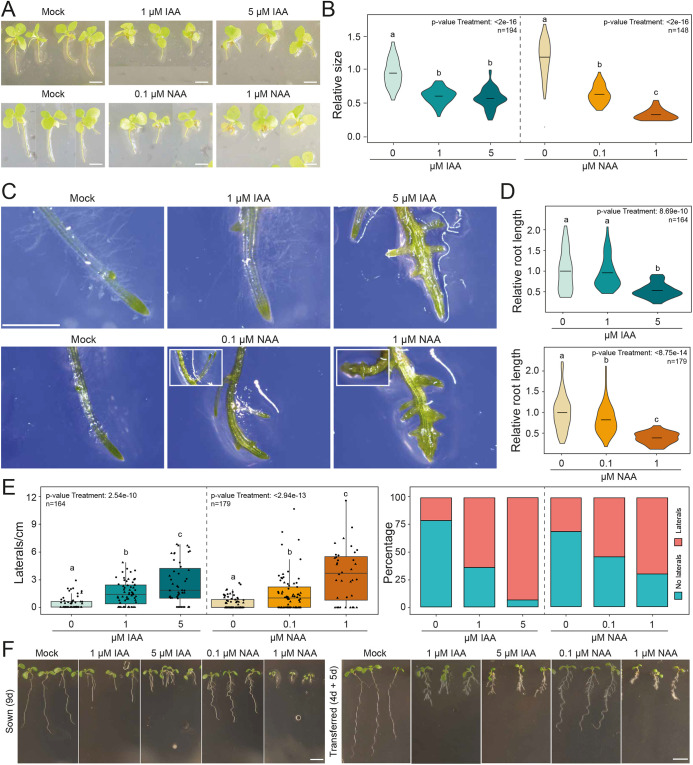
**Auxin responses in *Ceratopteris* sporophytes.** (A,B) Young sporophytes grown on various concentrations of IAA or NAA for 12 days (A) and quantification of root length (B). (C) Adventitious roots on sporophytes grown for 12 days in auxin-supplemented medium. Insets show unique double-splitting lateral root events. (D) Quantification of root length of adventitious roots grown on auxin with one representative replicate shown. (E) Quantification of lateral roots per cm of root and the frequency of roots bearing lateral roots under the different auxin conditions of two combined representative replicates. Box represents the IQR around the median value; whiskers represent most extreme data points or 1.5xIQR (maximum). (F) Representative images of *Arabidopsis* seedlings sown or transferred to auxin medium for the indicated times. Scale bars: 5 mm in A; 2.5 cm in C; 3 mm in F. Statistical significance is shown by letters based on ANOVA and Tukey's pair-wise comparison (*P*<0.05). The *P*-values for the data points calculated using ANOVA are indicated in the top right of B, D and E.

To test the responses of *Ceratopteris* sporophytes to external auxin, we transferred young sporophytes to medium containing different concentrations of IAA and NAA. In *Arabidopsis*, auxin is known to inhibit root growth while promoting branching (Fig. 2F) (Casimiro et al., 2001; Dubrovsky et al., 2008; Lavenus et al., 2013); however, previous research on *Ceratopteris* showed no response in branching (Hou et al., 2004)*.* To closely resemble *Arabidopsis* growth experiments, we initially focused on the growth and branching of the ‘embryonic’ root system. The ‘primary’ – first appearing – root showed a clear reduction in root growth at all IAA and NAA concentrations tested (Fig. 2A,B). One difficulty with *Ceratopteris* is the lack of a true primary root with its homorhizic root system. Therefore, responses were also measured on a mixed population of adventitious roots that showed a similar decrease in root growth upon auxin treatment (Fig. 2D). Auxin-treated roots also showed a clear increase of lateral root density upon auxin treatment. This increased branching was pronounced after 12 days of growth in auxin-supplemented medium (Fig. 2C,E), but is already visible after 3 days of growth (Fig. S3A,B). This increase of lateral density seems to be not only because of a reduction of growth or induced emergence of laterals, but also because of an actual induction or emergence of lateral roots. The absolute numbers of laterals per root under auxin treatment is higher than in control roots. Interestingly, the increased branching was not visible in the ‘primary’ root of the young sporophytes, clearly showing that branching is root age and/or stage-dependent, which is in line with the developmental plasticity of Ceratopteris roots (Hou and Hill, 2002). Hou et al. (2004) studied specifically and in much detail the 5th appearing adventitious root, and did not report an increase in lateral root formation. This seeming contradiction could be due to the fact that, here, a mixed pool of older adventitious roots (5th-10th) was studied and treatment times were longer.

The tissues that show responses to auxin in the *Ceratopteris* sporophyte and gametophyte are not homologous. Only rhizoids and root hairs are, genetically speaking, similar, as they seem to be specified by orthologous genes in non-homologous tissues (Jones and Dolan, 2012; Menand et al., 2007). We therefore analyzed the response to auxin in root hairs. Root hairs showed an increase in length and appear to initiate closer to the root tip (Fig. S3B,C) in auxin-treated roots. This induction of rhizoid growth on the roots resembles that on the gametophyte (Fig. 1D), but also that of *Marchantia* (Fig. 1E) and *Arabidopsis* roots (Fig. 2F). Thus, *Ceratopteris* roots show a combination of sporophyte-specific responses that are similar to those in *Arabidopsis* (Fig. 2F), and a response shared with the gametophyte in both *Ceratopteris* and *Marchantia*.

We next explored leaf development, as it represents a laminar morphology, which is also true for the gametophytic thallus. Notably, however, fern leaves are not direct homologs of angiosperm leaves and there is some debate on how many times leaves evolved independently in ferns (Boyce, 2005, 2010; Boyce and Knoll, 2002; Sanders et al., 2009; Tomescu, 2009), although their roots are regarded truly homologous structures compared to angiosperms (Pires and Dolan, 2012; Szövényi et al., 2019). One big advantage of *Ceratopteris* is that it makes compound leaves but also simple juvenile leaves (Conway and Di Stilio, 2020), very similar to *Arabidopsis*. Upon IAA treatment, no clear difference in leaf shape or growth was visible, which contrasts with gametophytes (Fig. 1). Inhibiting auxin export (e.g. with NPA) resulted in highly malformed leaves with altered patterns of vascular tissues, which partly resembles NPA-treated *Arabidopsis* leaves (Mattsson et al., 1999; Verna et al., 2015) (Fig. S4). NPA-grown *Ceratopteris* leaves had higher cardinality and connectivity indices than control leaves, suggesting that auxin transport inhibition leads to the formation of more veins that are more frequently connected and therefore that efficient auxin transport inhibits vein formation and connection. The effect of auxin transport inhibition was particularly striking in the primary embryonic leaf: whereas in control embryonic leaves the two veins branching off the single midvein remained unconnected, in NPA-grown embryonic leaves, the vein branches connected into a loop. Together, those observation suggest a conserved role for auxin transport in vein formation of tracheophyte megaphylls.

### Disrupting auxin transport can induce a sporophytic program

**Fig. 3.**
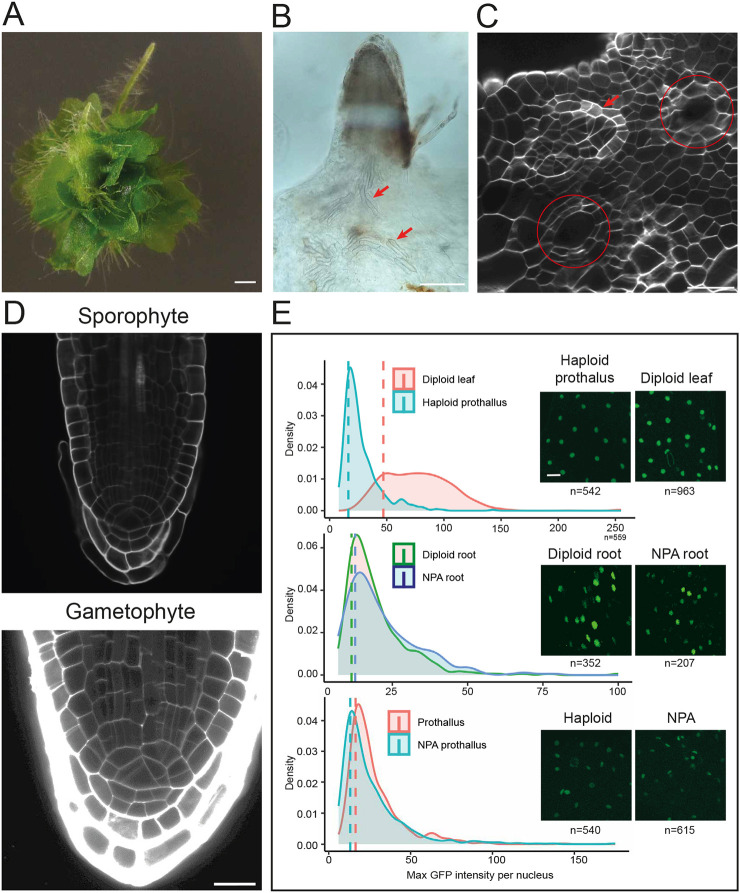
**Auxin-induced ectopic root formation in gametophytes.** (A) Phenotype of gametophytes grown on unsupplemented media for 25 days after transfer from media containing 5 µM NPA. (B) A cleared ectopic root shows that vascular elements (red arrows) that are not interconnected are formed in the gametophyte. (C) A cleared gametophyte showing the induction of a root apical cell (arrow) on prothallus tissue close to the archegonia (circles). (D) Comparison between sporophyte-derived roots and NPA-induced gametophyte-derived roots on a cellular level with both showing the distinct apical cell and root cap. (E) Ploidy analysis by quantifying GFP fluorescence intensities in the root between haploid thallus and diploid leaves, diploid sporophytic roots and NPA-induced roots, and haploid thallus and NPA-grown gametophytic thallus. The right images show examples of GFP patterns in the two conditions for each comparison. Scale bars: 1 mm in A; 200 µm in B; 50 µm in C; 50 µm in D; 40 µm in E.

When exploring the effect of auxin transport inhibition in gametophytes (Fig. 1F), we noticed that, in addition to the abnormal morphologies and increased rhizoid production, tissues also initiated root-like structures after prolonged treatment. These structures formed on the gametophytic thallus close to the notch meristem and produced root hairs (Fig. 3A). The anatomy of these structures was indistinguishable that of sporophytic roots (Fig. 3D). Additionally, these roots produced adventitious vascular-like tissue in the gametophyte, recognizable by the distinct spiraling secondary cell wall thickenings (Fig. 3B), resembling the description of cytokinin treated root callus in *Ophioglossum* (Peterson, 1969). Importantly, these roots did not originate from archegonia (Fig. 3C), as is the case for the apomictic *Dryopteris affinis* (Ojosnegros et al., 2024 preprint), or from sugar-induced apogamous haploid leaf-like structures (Cordle et al., 2007). Upon closer inspection, NPA-treated plants showed abnormal cellular morphologies close to archegonia, which mark the locations where root-like apical cells later appear and where roots grow out from (Fig. S5A). We interpret that these roots do not originate from an egg cell or zygote because: (1) they originate a few cells away from the archegonia; (2) they produce vascular tissues that are distributed randomly in the gametophyte and are not attached to a zygotic leaf (Fig. 3B); and (3) the roots develop much faster than zygotic roots (Fig. S5) (Aragón-Raygoza et al., 2020).

To verify the ploidy of the gametophyte-derived roots, a transgenic H2B-GFP expressing line (Geng et al., 2022) was imaged and the intensity of GFP fluorescence (Fig. 3E) was quantified as a proxy for ploidy. Sporophyte leaves and gametophytic thallus showed clearly distinct fluorescence intensities, consistent with their difference in ploidy (Fig. 3E), validating this approach for inferring ploidy. We found that sporophytic roots and NPA-induced gametophyte-derived roots showed no clear difference in GFP levels, indicating a similar (i.e. 2N) ploidy (Fig. 3E), in line with an additional quantification of DAPI fluorescence (Fig. S6A). Additionally, NPA-treated gametophytes showed no difference with mock gametophytes. Expression levels of the endogenous gene whose promoter was used to drive H2B-GFP expression (pCrHAM; Geng et al., 2022) were comparable between gametophyte and sporophyte tissues (Fig. S6B), suggesting that ploidy, not gene expression, creates the differences in H2B-GFP observed.

Consistent with NPA altering auxin distribution, NAA too could also ectopically induce roots on gametophytic tissue (Fig. S5B). The frequency of root initiation (Fig. S5C) was most prevalent upon releasing plants from NPA treatment, rising to 50% of plants developing ectopic roots. Keeping the plants continuously on NPA also resulted in ectopic root formation but only after long cultivation times (50 days). Together, this suggests that disrupting auxin maxima triggers the initiation of root primordia, but outgrowth happens only upon restoration of auxin transport. Interestingly, gametophytic root outgrowth happens at only ∼50 days after germination without medium transfer, explaining why this response was not observed previously (Withers et al., 2023b).

### Phylogeny and developmental phase conditions the transcriptional auxin response

Transcriptional responses to auxin show clear differences between three bryophytes (*P. patens*, *M. polymorpha* and *A. agrestis*) and two vascular plants (*C. richardii* and *A. thaliana*) (Mutte et al., 2018). There are two main differences: (1) the ratio of auxin-activated versus auxin-repressed genes is shifted towards activation in vascular plants, and towards repression in bryophytes; (2) the amplitude of auxin-activated gene expression is higher in vascular plants. Given that sporophyte tissue was sampled for the vascular plants and gametophyte tissue was sampled for the bryophytes, it is unclear which of these differences are due to phylogeny and which to developmental phase.

**Fig. 4.**
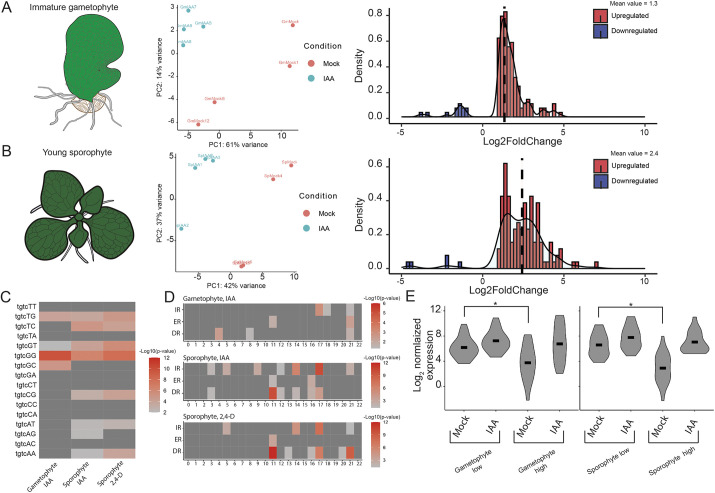
**Transcriptional auxin response of *Ceratopteris* gametophytes and sporophytes.** (A,B) Schematics of gametophyte (A) and sporophyte (B) stages on which the treatment was carried out (left), PCA plots showing the treatment effect (center) and density histograms of all DEGs (right; fold change of IAA/mock). Average value is indicated with dashed vertical line, upregulated genes are in red and downregulated genes are in blue. (C) The enrichment of 16 TGTCNN hexamers in auxin-responsive promoters for the two generations of differentially expressed genes from *Ceratopteris*. Color indicates the significance of enrichment, −Log10(*P*-value). (D) Abundance of TGTCNN repeats in auxin-responsive promoters of differentially expressed genes from *Ceratopteris*. *X*-axis shows the spacer length, *y*-axis indicates the fraction of genes with the repeats of specific structure. (E) Violin plots showing the RNA expression values of Log_2_ normalized expression values (DEseq2) for the 20 lowest and 20 highest DEGs in the gametophyte (left) and sporophyte (right). *P<0.005 (Wilcoxon-rank test).

We therefore compared transcriptional responses to auxin in both generations of *Ceratopteris*. We found that the amplitude of gene activation is higher in the sporophyte than in the gametophyte (Fig. 4A,B), suggesting that this difference is conditioned by the developmental phase. The sporophytic response appears to be robust between experiments, as it overlapped significantly with previously published data (Fig. S5A) (Mutte et al., 2018). By contrast, we found that in both generations, there is a dominance of gene activation, unlike in bryophytes (Fig. 4A,B). This suggests that this trait is defined by phylogeny. The shared DEG between the two generations show no clear distinction in their amplitudes (Fig. S7C), suggesting that this difference is partly due to the generation-specific targets.

A promoter analysis on the differentially expressed genes showed conserved enrichment of the high-affinity ARF binding AuxRE (TGTCGG) in both generations (Fig. 4C). ARFs bind AuxRE repeats cooperatively as homodimers (Boer et al., 2014; Korasick et al., 2014; Nanao et al., 2014), and we explored the syntax of such repeats in auxin-regulated genes in both generations. This revealed differential enrichment of spacing in tandem repeat motifs (Fig. 4D). The sporophyte motif closely resembled those motifs that were previously reported in *Arabidopsis* and maize (reviewed by Rienstra et al., 2023; Novikova et al. 2024 preprint). By contrast, the gametophyte-enriched motifs were weakly enriched, but distinct from the sporophyte-enriched motifs. This suggests that the targets are indeed discrete and might differ in their mode of activation, and that there is a sporophytic-tracheophyte specific motif spacing.

As is evident from the differential motif enrichment, there was only a small overlap in the genes controlled by auxin between the two generations (Fig. S7A). This overlap consisted of well-known auxin-inducible genes including Aux and IAA family genes, GH3 family genes, *YUCCA* and EXPANSIN family genes (Fig. S8A-C). Upon ortholog grouping and comparison, these genes are shared both with *Marchantia* and *Arabidopsis*. However, there was no clear enrichment of overlap between *Arabidopsis* and *Ceratopteris* sporophytes, or *Marchantia* and *Ceratopteris* gametophytes (Fig. S8A-C). Thus, we could not define clear shared sporophytic or gametophytic auxin-regulated genetic programs.

The amplitude of gene activation is defined by expression level in the absence and presence of auxin. High amplitude can therefore be generated by efficient repression in the absence of auxin, or by effective activation in its presence. We explored the likely mechanism underlying the difference in amplitude between gametophyte and sporophyte. The difference between highly and lowly upregulated genes was clearly due to an increase in repression in both generations (Fig. 4E). By comparing the two generations, we found that the 20 most strongly activated genes in sporophytes show a lower expression (log2=2.76 normalized expression) under mock conditions than in gametophytes (log2=3.78 normalized expression), whereas their expression upon auxin treatment was more similar between generations (log2=6.75 normalized expression versus log2=7.34 normalized expression). This suggests that the increased amplitude of gene activation in the sporophyte is caused by efficient repression.

### Aux/IAA expression levels condition auxin sensitivity

We found that *Ceratopteris* gametophytes respond to auxin in a manner similar to the *Marchantia* gametophyte. Transcriptional responses are a direct outcome of the components of the nuclear auxin pathway (NAP) encoded by the genome of each species and expressed in each tissue. To identify possible genetic drivers of differences between the gametophyte and sporophyte auxin response, we explored expression patterns of NAP components. The core components are: (1) the DNA-binding auxin response factors (ARFs), which are divided into auxin-dependent activating (A class) and auxin-independent repressing (B class); (2) Aux/IAA repressors; and (3) TIR1/AFBs that promote Aux/IAAs degradation in the presence of auxin (Lavy and Estelle, 2016). We found that Aux/IAA family genes are more prominently expressed in the *Ceratopteris* sporophyte (Fig. S9). This analysis was further extended to publicly available transcriptomes from *Ceratopteris* across more developmental stages (Marchant et al., 2019, 2022), showing a similar pattern (Fig. S10). This is consistent with an increased ability to repress target genes in the absence of auxin. However, A-class ARFs also seemed more abundant in the sporophyte generation, possibly also explaining the difference in response. B-class ARFs showed a less clear pattern or were very lowly expressed (Fig. S9), and it is unclear what the biological significance of changes in expression at such low expression levels is.

**Fig. 5.**
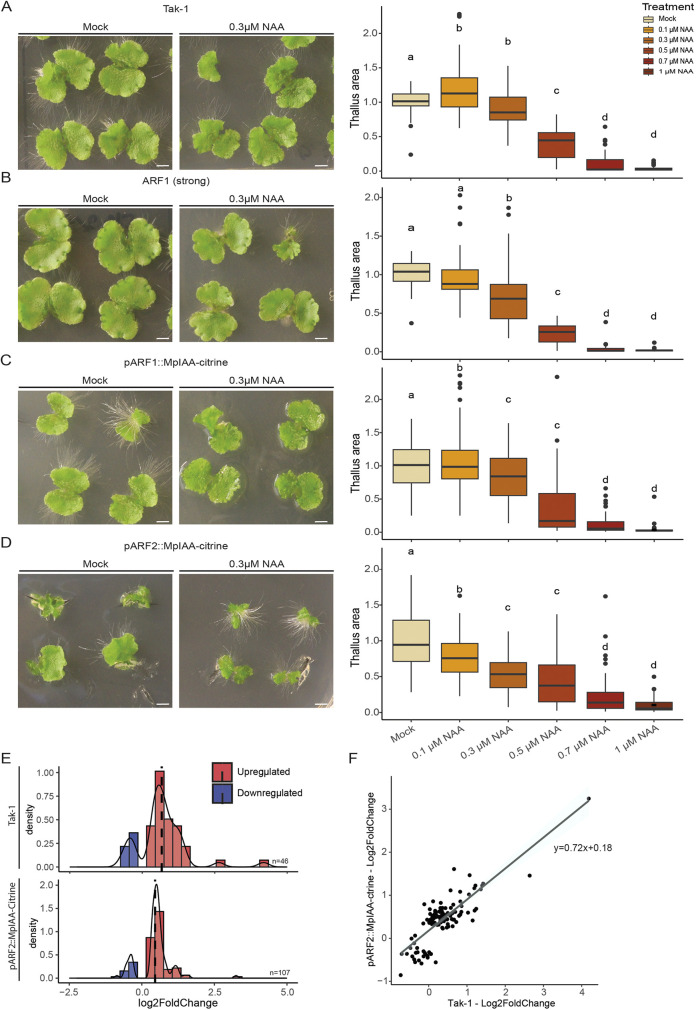
**Modulating auxin response characteristics in *Marchantia*.** (A-D) Tak-1 wild-type (A), pARF1-ARF1 (B), pARF1-MpIAA (=relative weak expression) (C) and pARF2-MpIAA (=relative strong expression) (D) gemmalings grown on mock media and 0.3 µM NAA (left panels), and quantification of relative projected thallus area of three combined replicates for each of the genotypes and across a range of NAA concentrations (right panels). Box represents the IQR around the median value; whiskers represent most extreme data points or 1.5xIQR (maximum). (E) RNA-seq of *Marchantia* pARF2:MpIAA and Tak-1 density histograms of auxin-responsive genes (*P*_adj_<0.05, Log_2_Foldchange(>0.3). (F) Expression values of all auxin-responsive genes in either of two genotypes and the linear trendline describing the point cloud. Scale bars: 2.5 mm. Statistical significance is shown by letters, based on ANOVA and Tukey's pair-wise comparison (*P*<0.05).

Our transcriptomic data, along with gene content and differential expression of NAP components support a model where differential Aux/IAA expression and/or A-class ARF expression between generations creates distinct potential for high-amplitude gene activation. Indeed, relative to *Marchantia*, *Ceratopteris* has undergone duplications both in the Aux/IAA family and the A-class ARF subfamily (Mutte et al., 2018). To directly test the contribution of A-ARF or Aux/IAA copy number to auxin responsiveness, we generated transgenic *Marchantia* lines that either had slightly increased expression (Log2=4) of a mCitrine-tagged copy of MpARF1 (A-class ARF) or that express an additional copy of MpIAA, under the MpARF2 or MpARF1 promoter. Increased MpARF1 expression was achieved by complementing the *arf1-4* loss-of-function mutant (Kato et al., 2017) with a tagged, transgenic copy of the wild-type protein, and by selecting a line that had increased *ARF1* expression compared with wild-type plants (Fig. S11B). The advantage of *Marchantia* is the relatively simple system of only one A-class ARF and one Aux/IAA (Flores-Sandoval et al., 2015; Kato et al., 2020). Transgenic plants were tested for their auxin responsiveness by measuring thallus growth inhibition by a range of auxin concentrations (Fig. 5A-D). Increased MpARF1 levels did not induce a clear difference in auxin response compared with Tak-1 wild type (Fig. 5B). By contrast, lines expressing an additional copy of MpIAA under the MpARF2 promoter showed a difference in overall phenotype and an increase in auxin sensitivity compared with Tak-1 (Fig. 5D). Like the endogenous MpIAA protein (Das et al., 2024), the additional copy was undetectable in control treatments in transgenic lines, but accumulated upon inhibition of the proteosome (Fig. S11A).

The increased auxin responsiveness in lines expressing an additional Aux/IAA copy is consistent with our predictions, but may or may not reflect a higher amplitude in auxin-dependent gene activation. To directly test the properties of gene expression output, we performed RNA-seq upon auxin treatment on a representative *proMpARF2-MpIAA-Citrine* line. We did not detect a clear shift in response amplitude of gene activation between Tak-1 and *proMpARF2-MpIAA-Citrine* plants (Fig. 5E,F). We used non-stringent filtering of the differentially expressed genes (*P*adj<0.05; Foldchange>1.25) to identify differentially expressed genes, despite the low-inductive nature of auxin responses in *Marchantia*. With this filtering, a substantially larger group of genes is differentially expressed in the MpIAA-Citrine lines compared with Tak-1 (Fig. 5E), suggesting increased responsiveness. Upon plotting only the expression of genes that are auxin-responsive in the MpIAA-Citrine line, a slight increase in repression was visible [Log2(TPM)=8.93 instead of 9.25 in Tak-1]. However, this was also accompanied by an increase in activation (9.7 versus 9.37; Fig. S11C), showing a mixed response on a transcriptional level. Based on these experiments in *Marchantia*, we conclude that Aux/IAA expression level does increase the degree of auxin responsiveness, but that the sporophyte-like increased transcriptional response amplitude is likely caused by factors beyond Aux/IAA dose.

### Conserved rapid auxin responses in *Ceratopteris*

**Fig. 6.**
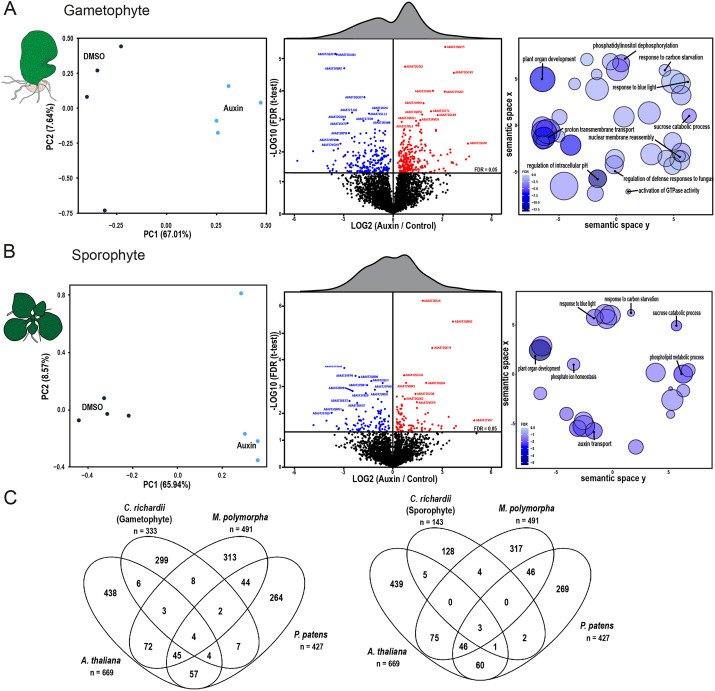
**Auxin-triggered protein phosphorylation in gametophyte and sporophyte generations.** (A,B) PCA analysis (left), differential phosphorylation (center) and GO analysis of differentially phosphorylated proteins (right) after 2 min of treatment with 100 nM IAA in *Ceratopteris* gametophytes (A) and sporophytes (B). (C) Overlap of phosphosite orthogroups between the *Ceratopteris* gametophytic prothallus and young whole sporophytes, and previously reported datasets for *Marchantia* thallus, *Physcomitrium* gametophores and *Arabidopsis* roots from Kuhn et al. (2024).

Besides transcriptional responses, auxin promotes a number of fast cellular responses, including cytoplasmic streaming and altered proton transport across the plasma membrane (Ayling and Clarkson, 1996; Barbez et al., 2017). We recently found that some of these responses are mediated by proteome-wide rapid auxin-triggered protein phosphorylation involving a conserved RAF-like protein kinase (Kuhn et al., 2024). Phosphoproteomic profiling in *Arabidopsis*, *Physcomitrium* and *Marchantia* showed conservation of the response, and identified sets of common targets, as well as bryophyte-specific and species-specific targets. Although gametophytes were used for the two bryophyte species, it is hard to tell which of these differences are due to phylogeny and which is due to ontogeny. We therefore performed phosphoproteome profiling upon 2 min of treatment with 100 nM of IAA in both gametophytes and whole sporophytes in *Ceratopteris*, to compare entire plants with each other. Auxin triggered differential protein phosphorylation in both generations, but the number of auxin-regulated phosphotargets was modest when compared with *Arabidopsis* or bryophyte species. This may in part be due to the fact that we sampled entire sporophytes to make a fair comparison with the whole gametophyte, whereas, for *Arabidopsis*, only roots were sampled. When comparing the two generations in *Ceratopteris*, we did not find clear differences in the profile of phosphorylation (Fig. 6A,B). Auxin-regulated phosphorylation in both generations more closely resembled that in bryophytes, than in *Arabidopsis*. Both shared and unique functions are targeted by auxin-triggered phosphorylation in the two generations (Fig. 6A,B). Among these shared GO-terms (‘plant organ development’, ‘carbon starvation’ and ‘response to blue light’), at least one is conserved across all species that were previously tested (Kuhn et al., 2024).

We next explored the overlap between phosphotargets between the two generations, and with *Arabidopsis*, *Marchantia* and *Physcomitrium*. In general, the overlap is limited (Fig. 6C) yet of the same order as overlap between the different species tested by Kuhn et al. (2024). The estimated divergence times of the tested species used here is ∼500 million years (Donoghue et al., 2021). Given these enormous evolutionary distances, there is substantial sequence divergence within protein families and large differences in gene family sizes that makes establishing direct relationships very challenging. Additionally, we compared *Marchantia* thallus, *Physcomitrum* gametophores and *Arabidopsis* roots from Kuhn et al. (2024) with *Ceratopteris* prothallus and *Ceratopteris* whole sporophytes, which are morphologically very distinct tissues. Owing to the morphologically different tissue sampled, the observed overlap and conservation is likely an underestimation. This suggests that, apart from a deeply conserved auxin-sensitive core, a wide range of species-specific and generation-specific changes are induced. Thus, auxin-triggered phosphorylation is conserved in *Ceratopteris*, and patterns of response are conditioned both by ontogeny and phylogeny.

## DISCUSSION

Here, we describe a set of different auxin responses in the model fern *Ceratopteris* across its two indeterminate multicellular generations. On a phenotypic level, we see that gametophytes generally resemble thalloid bryophytes like *Marchantia*, while sporophytes mostly resemble flowering plants like *Arabidopsis*, in their capacity to respond to auxin. Auxin responses are numerous (Paque and Weijers, 2016), and it remains to be tested whether the same patterns of response analogy hold for other growth or developmental processes. This similarity between *Marchantia* and *Ceratopteris* gametophytes is striking but is mirrored by the developmental homology with the same set of organs and cell types formed (i.e. rhizoids, antheridia and archegonia). One could even argue that *Ceratopteris* gametophytes are simpler than *Marchantia* gametophytes due to their short-lived nature and lack of a proper *z*-axis development with no air chambers or storage tissue as in *Marchantia* (Conway and Di Stilio, 2020). Similarly, it appeals to reason that *Ceratopteris* sporophytes resemble *Arabidopsis* seedlings in their response to auxin, given that these species share the same evolutionary origin of their roots and vasculature (Szövényi et al., 2019). It is intriguing though that the response to auxin response inhibition in *Ceratopteris* leaves resembles that in *Arabidopsis* leaves, which evolved independently from each other (Pires and Dolan, 2012; Tomescu, 2009). Both are vascularized shoot branches, but the lamina connecting the veins evolved independently, as did their minor veins and closed loops (Boyce and Knoll, 2002; Doyle and Hickey, 1976; Galtier, 2010; Paliwal et al., 1976; Sanders et al., 2007), which makes it surprising that both depend on polar auxin transport. Other experiments showed that polar auxin is necessary for spike and/or frond elongation and uncurling (Briggs and Steeves, 1959; Peterson and Cutter, 1969; Steeves and Briggs, 1960; Voeller, 1960; Wetmore and Pratt, 1949); however these compound fronds are difficult to compare with *Arabidopsis* simple leaves or with the juvenile leaves of *Ceratopteris*.

Long-term treatments with auxin or auxin transport inhibition in *Ceratopteris* gametophytes showed that sporophytic organs can be initiated, demonstrating the power of auxin as a developmental signal. To our knowledge, this is first report of such transdifferentiation, although it bears a superficial resemblance to the initiation of microspore-derived embryos in some flowering plant species (Corral-Martínez et al., 2020; Supena et al., 2008). A plausible scenario is that auxin triggers (epigenetic) reprogramming to a diploid state from which sporophytic organs can emerge. Genetic studies in bryophytes identified such two mechanisms to induce the sporophytic program: (1) mutants in the Polycomb repressive complex 2 in *Physcomitrium* develop branching sporangia-like structures (Okano et al., 2009) with conserved functioning in rice (Wu et al., 2023); and (2) the diploid zygotic program in *Marchantia* seems to depend on the activation of KNOX/BELL TALE-homeodomain proteins (Dierschke et al., 2021; Hisanaga et al., 2021). Evidence from rice suggests that these pathways share a common nominator in the PRC2-associated coiled-coil protein (PACP) (Tan et al., 2022). Ultimately, these pathways lead to epigenetic reprogramming by histone modification. Therefore, it is likely that our NPA treatments alter the epigenetic state and thereby induce a sporophytic programme, whether it depends equally on PRC2 and/or KNOX/BELL remains to be determined.

Similar to the NPA-induced roots we observed, it has previously been reported that ‘rod-like structures’ develop from regenerating sporophytic callus in *Ceratopteris* (Xiao and Li, 2024). Similarly, roots are induced from eudicot callus when treated with a high ratio of auxin over cytokinin (Che et al., 2006) and *Arabidopsis* root meristems show high signals of the auxin transcriptional reporter Dr5v2 (Liao et al., 2015). In line with the capacities of both NPA and NAA to induce ectopic roots on *Ceratopteris* gametophytes, it seems that auxin maxima play an important role for the transdifferentiation. Indeed, growth experiments on L-Kynurenine, an auxin biosynthesis inhibitor, did not result in similar phenotypes (Withers et al., 2023b). Others have reported that sugar in the growth media could induce apogamy in *Ceratopteris* gametophytes (Bui et al., 2012, 2017). As ectopic root formation did not depend on sugar, we interpret that these structures are derived from a process distinct from apogamy. Identifying the intermediate stages and associated gene expression changes could help in identifying core root specification genes, as well as in the identifying sporophyte signature genes.

We also find that the amplitude of auxin response is stronger in the sporophyte than in the gametophyte, reflecting the bryophyte-tracheophyte split, and suggesting this is an emergent property of the developmental generation. It is unlikely that differences in tissue permeability to auxin contribute to these different responses, as *Ceratopteris* gametophytes are less complex in architecture, and most cells are directly exposed to the media. However, PINs (auxin efflux carriers) are less expressed in the gametophyte (not shown) and important for sporophytic development in *Ceratopteris* (Xiang and Li*,* 2024), which may dampen the response. One caveat here is the lack of bryophytic sporophytes in our study, which prevents us from drawing firmer conclusions about the ancestral state. There are interesting reports of the role of auxin in bryophytic sporophyte development showing similarities to tracheophyte sporophytes, but they lack information about the genetic targets (Bennett et al., 2014; Fujita et al., 2008; Poli et al., 2003; Schnepf et al., 1979). Similarities also exist between *Physcomitrium* gametophores and tracheophyte shoots in, for example, the regulation of shoot branching (Coudert et al., 2015; Landberg et al., 2021; Thelander et al., 2022). However, *Physcomitrium* gametophores have a completely different growth habit from *Ceratopteris* gametophytes, and whether the ancestral state of the gametophyte is a thallus or leafless axis remains unknown (Bowman, 2022).

We tested directly whether the inferred duplications in A-class ARFs and Aux/IAAs that preceded the emergence of ferns may contribute to sporophyte-like response dynamics. Our results show that growth indeed becomes more sensitive to auxin when an additional Aux/IAA copy is expressed in the *Marchantia* gametophyte. However, gene expression does not fully resemble that in sporophytes of *Ceratopteris* or *Arabidopsis* in terms of amplitude. This result can be interpreted in many ways, but it is clear that there is additional complexity in the genetic architecture of the NAP and its differences between generations. A logical next step would be to combine the expression of an additional A-ARF copy with an additional Aux/IAA copy or move to a more-complex redundant system like *Ceratopteris*, with many ARFs and Aux/IAAs. Further advancements in facile genome editing in *Ceratopteris* (Jiang et al., 2024 preprint; Xiang and Li, 2024), silencing (Bui et al., 2017; Withers et al., 2023a) and transgene expression (Geng et al., 2022; Plackett et al., 2014) will also help to further explore the genetic requirements for auxin response in both generations.

The above-mentioned similarities between Ceratopteris gametophytes and bryophytes suggest a conserved mechanism of auxin response based on their generation. However, the targets of the auxin response are very divergent, besides the small core sets, as shown by orthogroup comparisons for both transcriptomics and phosphoproteomics. This is probably due to their distinct phylogenetic placement and 500 million years of divergence (Donoghue et al., 2021) but also likely an underestimation due to the crude comparison between highly divergent tissue morphologies. Together with the previous points, this suggests that the exact targets of the response are strongly dependent on phylogeny, while the nature and mechanisms of the response depend more on developmental phase.

In summary, by studying the fern *Ceratopteris richardii* and through comparisons with *Marchantia* and other species, we have identified the developmental phase as a major contributor to the properties of the auxin response. This helps further understand the divergence but, equally importantly, the homology in hormone signaling between land plants. We expect that further exploration of the two generations in *Ceratopteris* will shed light on gametophyte and sporophyte developmental programs, and on their origin and homology.

## MATERIALS AND METHODS

### Plant growth conditions

Spores of *Ceratopteris richardii* strain Hn-n (Hickok et al., 1995) were sterilized and grown as described previously (Plackett et al., 2014) in a Hettich MPC600 plant growth incubator set at 28°C, with 16 h of 100 μmol m^–2^ s^–1^. Plants were grown on ½-strength MS medium supplemented with 1% sucrose, unless stated otherwise. Gametophytes were grown from spores and synchronized by imbibing the spores in the dark in water for more than 4 days. Sporophytes were obtained by flooding plates containing sexually mature gametophytes with water. Sporophytes used for imaging were around 3-4 weeks old. *Marchantia polymorpha* plants were grown on ½-strength Gamborg's B5 medium at 22°C with constant light. *Arabidopsis thaliana* plants were grown on ½-strength MS with 1% sucrose at 20°C, 60% humidity with 16 h of light/day.

### Auxin growth experiments

For gametophytes, spores were sown directly on plates supplemented with indole-3-acetic acid (IAA; Alfa aeser) or 1-naphthaleneacetic acid (NAA, Sigma). Size measurements were carried out when plants reached sexual maturity (±6/7 days). Alternatively, germinated spores were transferred onto auxin-supplemented medium before the lateral notch meristem was established (±5 days) and grown for another 5 days. N-1-naphthylphthalamic acid (NPA, Sigma) treatments were carried out in a similar manner. Gametophytes were imaged with a Leica M205FA epifluorescence microscope and their size was quantified by measuring their length and width. Sporophytes were grown in liquid ½-strength MS without sucrose for root phenotyping. For every individual experiment, young sporophytes from the same plate were used to synchronize their development as much as possible. Sporophytes were grown for 12 days to measure root growth and branching. Sporophytes were imaged with a Canon EFS (18-135 mm) camera, a close-up of the root tips was captured using a Leica M205FA epifluorescence microscope. Root lengths were measured using ImageJ and scored manually for the number of lateral roots. Rhizoid images were taken 3 days after transfer. *Marchantia* and *Arabidopsis* plants were treated in a similar manner, except that the medium was different, as described in the previous section.

To induce sporophytic roots on gametophytic thallus, spores were grown for at least 10 days on ½-strength MS containing 1% sucrose and 5 µM NPA; afterwards, they were transferred to NPA-free medium. The first roots appeared 25 days after transfer. Sporophytic roots were also obtained when gametophytes were grown for 50 days on 5 µM NPA or if germinated normally and then transferred to 5-10 µM NPA or 5-10 µM NAA.

### Leaf venation analysis

NPA-treated leaves were harvested 2 weeks after young sporophytes were transferred to medium containing 10 or 20 µM NPA. Only the youngest developed leaves were harvested to ensure that leaf primordia developed under NPA conditions. Leaves were fixed and cleared in ethanol:acetic acid for at least 1 day. Afterwards, leaves were rehydrated in 70% ethanol and stored at 4°C until imaging. Imaging was carried out using a Leica M205FA epifluorescence microscope. Quantification was carried out similar to Verna et al. (2015). Briefly, the number of touch, end, break and exit points of the veins were calculated for every leaf. From those numbers, the connectivity, absolute cardinality and continuity index were calculated. The relative indexes were used to enable the pooling of different experiments. Statistical analysis was carried out by first testing for equal variance with a F-test and then using an unpaired Student's *t*-test to test for equal means.

### Ploidy analysis

CrHAM::H2B-GFP line spores have been described by Geng et al. (2022) and were grown based on a previously described method (Plackett et al., 2014). GFP intensities were imaged with a Leica SP5 confocal microscope, with HyD detectors on photon counting mode to facilitate quantification. *Z*-stacks were obtained throughout the whole tissue and maximum projections of those stacks were subsequently quantified in ImageJ. Nuclear intensity was quantified by first selecting the nuclei by binarizing the image. By subsequently analyzing the particles, all nuclear regions of interest (ROIs) could be selected, which were then imported to the original image to measure their intensities. All ROIs smaller than a given size (arbitrary area<30) were discarded as they were outside the expected size range for nuclei. DAPI quantification was carried out in a similar manner by staining cleared roots (Kurihara et al., 2021) overnight with 50 µg/µl DAPI and washing once before imaging.

### RNA isolation and sequencing

For RNA isolation, immature gametophytes were grown for 5 days on a 100 µm nylon mesh. Young sporophytes of 22 days after fertilization were transferred to a new plate with a 100 µm nylon mesh and collected 5 days later, after which new roots had developed. At this stage, sporophytes had approximately five leaves. Auxin treatments were performed by dissolving an IAA stock in liquid ½-strength MS with no sugar to a final concentration of 1 µM IAA or a DMSO control. The medium was preheated to 28°C to prevent any cold shock on the plants. Plates were taken out of the incubator, flooded with IAA or DMSO and returned to the incubator for 1 h.

*Ceratopteris* RNA was isolated with the Qiagen RNeasy kit and Total RNA was treated with RNase-free DNase I set (Qiagen). RNAseq libraries were prepared and up to 20 million 150 bp paired-end sequences were collected by Illumina-sequencing by Novogene (UK). RNA quality was checked using FastQC (www.bioinformatics.babraham.ac.uk/projects/fastqc), reads were mapped by Salmon, the obtained raw read counts were normalized and differentially expressed genes (*P*_adj_<0.05) were identified using DEseq2. All plots were made using ggplot2 (https://cran.r-project.org/web/packages/ggplot2/index.html) besides the upset plot with UpSetR (https://cran.r-project.org/web/packages/UpSetR/index.html). The RNAseq raw reads have been deposited in the NCBI Short Read Archive (SRA) under the BioProjectID PRJNA1149654.

For *Marchantia*, gemmae were grown for 9 days at 22°C. IAA treatments were carried out as described previously by Kuhn et al. (2024). Briefly, before IAA treatment, plants were flooded with liquid medium overnight before incubating with 1 µM IAA for 1 h. Marchantia RNA was isolated with the Qiagen RNeasy kit with an additional Trizol step before column binding. RNAseq libraries were prepared and up to 20 million 150 bp paired-end sequences were collected by Illumina-sequencing by BMKGENE (Germany). RNA quality was checked using FastQC (www.bioinformatics.babraham.ac.uk/projects/fastqc), reads were mapped by Hisat2, the obtained raw read counts were normalized and differentially expressed genes (*P*_adj_<0.05) were identified using DEseq2. The RNAseq raw reads have been deposited in the NCBI Short Read Archive (SRA) under the BioProjectID PRJNA1149659.

### Expression analysis

For the comparison between gametophytes and sporophytes, the normalized expression values of the mock treatments of both life stages were compared using R package ComplexHeatmap (https://bioconductor.org/packages/release/bioc/html/ComplexHeatmap.html). Expression values across all developmental stages were retrieved from Marchant et al. (2022) and TPM values were normalized to a Z-score and plotted with ComplexHeatmap.

### Promoter analysis

We used the reference genome of fern *Ceratopteris richardii* v2.1 from Phytozome DB. Promoters of all genes were analyzed for over-representation of hexamers in the interval 600 bp upstream from the transcription start site (TSS) to either the translation start site or 1 kb downstream of the TSS, depending on which was smaller. We analyzed three sets of upregulated DEGs (foreground sequences) and generated for each of them a background consisting of the same genomic intervals as in the resting genes. First, for each pair of ‘foreground versus background’ sets, we used Fisher's exact test to estimate the enrichment for TGTCNN consensus sequences. Here and below, this test counted the number of genes. Second, we applied the package MCOT (Levitsky et al., 2019) to count the numbers of genes containing in the genomic interval TGTCNN repeats with spacers from 0 bp to 25 bp, and specific orientations [direct (DR), inverted (IR) and everted (ER) repeats]. For each pair of ‘foreground versus background’ results and for any possible mutual location and orientation of hexamer in pairs, we estimated the enrichment of their co-occurrence using Fisher's exact test.

### Orthogroup analysis

Orthogroups between the different species were identified using Orthofinder (Emms and Kelly, 2019). Therefore, transcriptomes from the species used, i.e. *Arabidopsis thaliana* (Araport11), *Marchantia polymorpha* (v6.1) and *Ceratopteris richardii* (v2.1), were used and common orthologous sequences were identified. Similarly, for the phospho-proteomics, orthogroups were identified from the proteomes of the different species. The auxin-responsive DEG or phosphosites from the different species were converted to their specific orthogroup and subsequently overlaid using a Venn diagram.

### Plasmid construction

MpARF1 and MpARF2 promoters (+3 kb upstream) were amplified with the primer set HK120/HK125 and HK126/HK127, respectively (Table S1), and cloned into pMPGWB307 using the Xbal site (pHKDW031/038). MpIAA and MpARF1 CDS were subcloned into pENTR/D (Thermo Fisher Scientific) using the primer combinations of MpIAA_entry/JHG081 and HK009/HK015, respectively. These genomic CDS sequences were then transferred to the pHKDW031 (pARF2) or 038 (pARF1) with Gateway LR Clonase II Enzyme mix (Thermo Fisher Scientific). pHKDW038 has been described previously and was kindly provided by Hirotaka Kato (Kato et al., 2017, 2020).

### RT-qPCR

MpARF1 expression was validated in multiple complemented arf1-4 mutant (Kato et al., 2017) to select for a higher expressing line. RNA was isolated from 10-day-old gemmalings as described previously (see RNA isolation and sequencing section). 1 µg of total RNA was used for cDNA synthesis (iScript cDNA synthesis kit, Bio-Rad) according to the manufacturer's instructions. RT-qPCR was performed using a 384CFX Connect Real-Time PCR Detection system (Bio-Rad) and iQ SYBR Green Supermix (Bio-Rad). A two-step cycle of 95°C for 10 s followed by 60°C for 30 s was repeated for 40 cycles, followed by a melt-curve analysis. Three biological and two technical replicates were used. All primers used are listed in Table S1. The geometric mean of MpSAND, MpAPT7 and MpAPT3 (Saint-Marcoux et al., 2015) was used to normalize expression of MpARF1 according to Taylor et al. (2019).

### *Marchantia* transformation

A protocol based on the *Agrobacterium*-mediated transformation of *M. polymorpha* regenerating thalli (Kubota et al., 2013) was used. Briefly, Agrobacterium cultures were grown for 2 days in liquid LB medium. Afterwards they were spun down and resuspended in liquid Gamborg medium supplemented with sucrose and casamino acids and acetosyringone, and left for 6 h. Tak-1 thallus was cut into 1 mm×1 mm pieces and added to liquid medium together with Agrobacterium. Co-cultures were grown for 3 days at 22°C while shaking. After washing, positive transformants were selected on medium containing chlorsulfuron (0.5 µM) and cefotaxime (100 mg/l). Transformants were validated by PCR and microscopy. The strong expressing line for ARF1 was validated by qPCR

### Phosphoproteomics

*Ceratopteris* gametophytes and sporophytes were grown on mesh and treated for 2 min with 100 nM IAA and harvested immediately. Protein purification and phospho-peptide enrichment and measurements were carried out as described previously (Kuhn et al., 2024). The mass spectrometry proteomics data have been deposited in the ProteomeXchange Consortium via the PRIDE (Perez-Riverol et al., 2022) partner repository with the dataset identifier PXD054985.

### Confocal microscopy

Ceratopteris gametophytes grown on auxin-supplemented medium were cleared and fixed using Clearsee alpha (Kurihara et al., 2021), and stained with SR-2200/Renaissance (Musielak et al., 2016). Roots were cleared and stained in a similar manner. Imaging was carried out using a Leica SP5 confocal microscope.

Marchantia MpIAA-Citrine was detected with a Leica SP8 confocal microscope. MG132 (Sigma) treatments were carried out on gemmae that were allowed to germinate for 8 h in the presence of MG132 and imaged afterwards.

## Supplementary Material



10.1242/develop.203026_sup1 Supplementary information
