# Supplementary material for: Analysis of auxin responses in the fern Ceratopteris richardii identifies the developmental phase as a major determinant for response properties
Source: Development. 2024 Sep 26;151(20):dev203026. doi: 10.1242/dev.203026 (PMC11449451; doi:10.1242/dev.203026)
Supplement: Supplementary information [file develop-151-203026-s1.pdf]

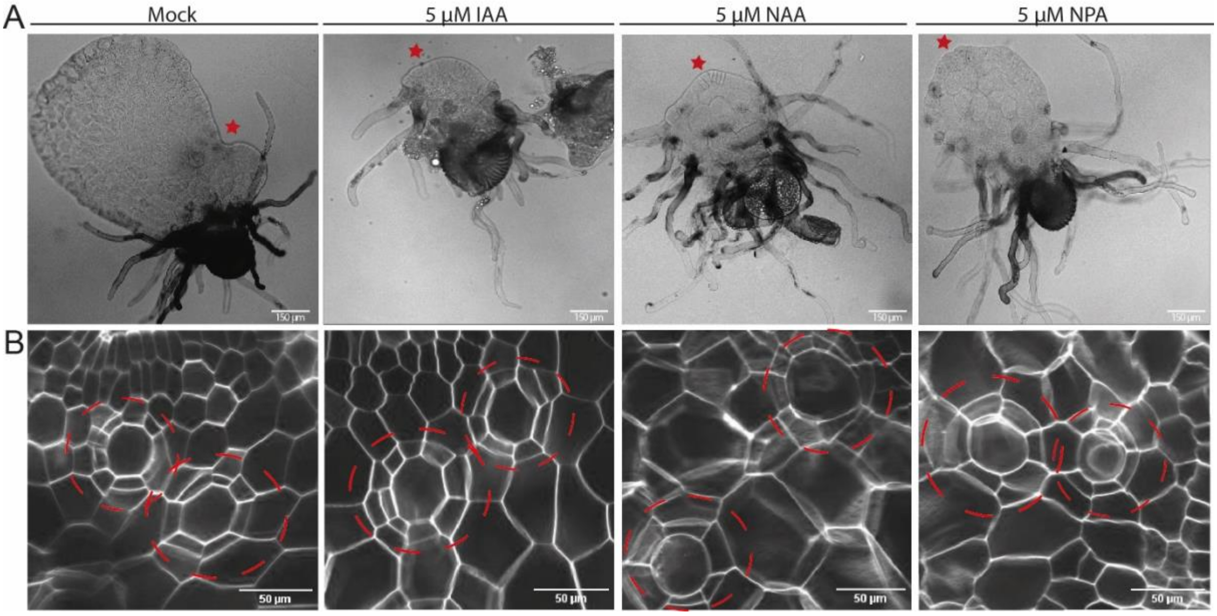

Fig. S1. Notch and archegonia phenotypes in gametophytes upon auxin treatment, showing representative pictures. A) Gametophytes moved to hormone supplemented medium after 4 days. B) Confocal image of mid-plan through mature archegonium of the same gametophytes as shown in A. Dashed lines encircle archegonia, red asterisks point at notch meristem locations. Scale bars upper panels are 150 µm, bottom panels 50 µm.

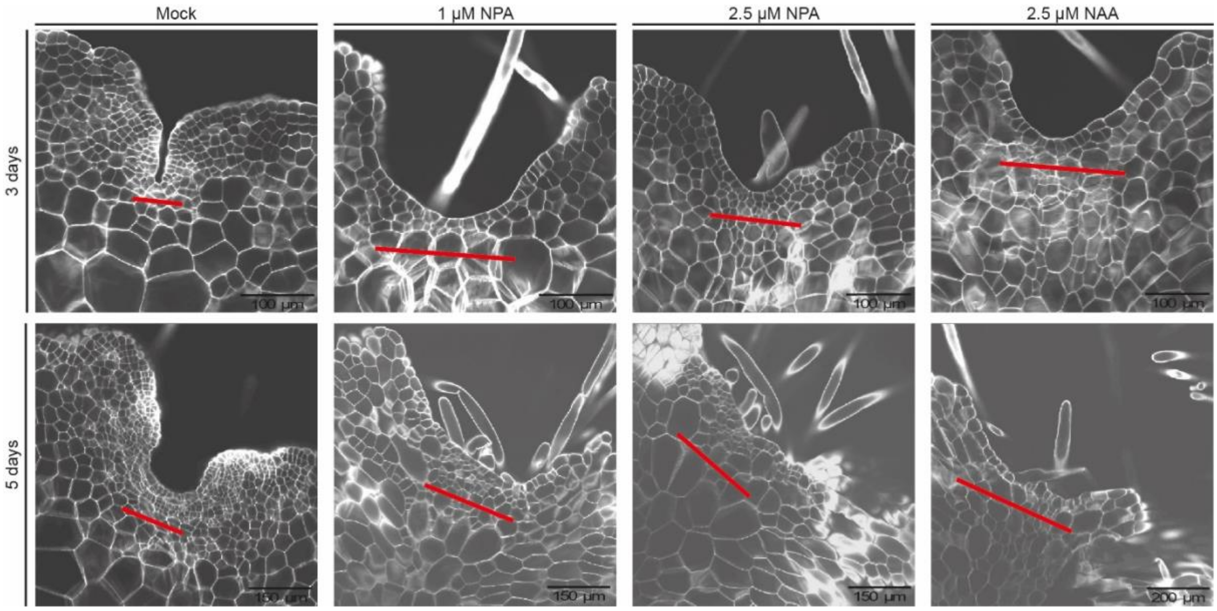

Fig. S2. Apical notch phenotypes in Marchantia. Gemmae grown for 3 days (upper panels) or 5 days (lower panels) under different auxin treatments. showing representative pictures. Scale bars are depicted in pictures.

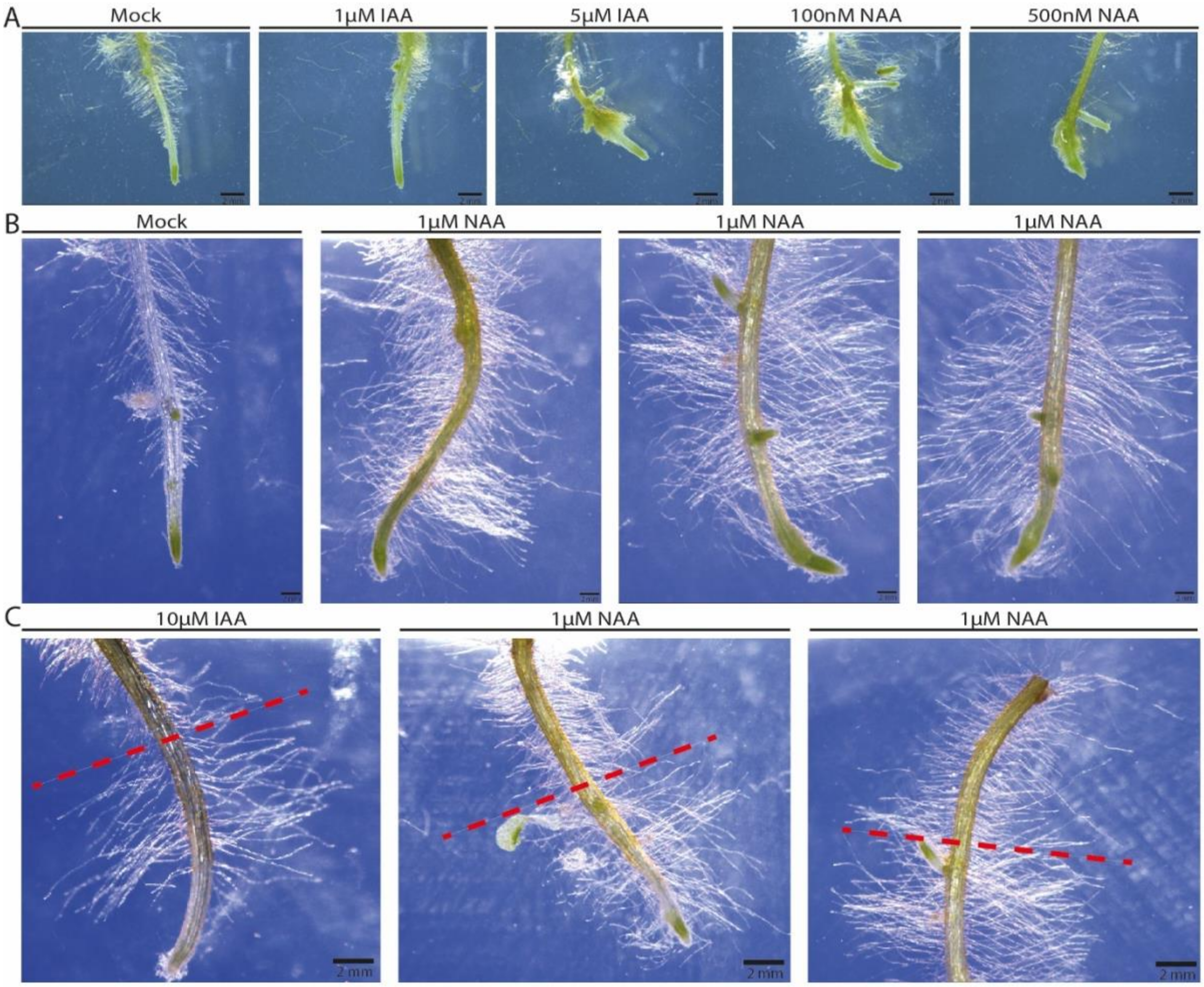

Fig. S3. Root hair response to 3 days of auxin treatments. A) The induction of laterals is visible while B) at the same time, root hairs seem to increase their length and abundance C) especially clear below the red dashed line. Scale bars are 2mm in all panels.

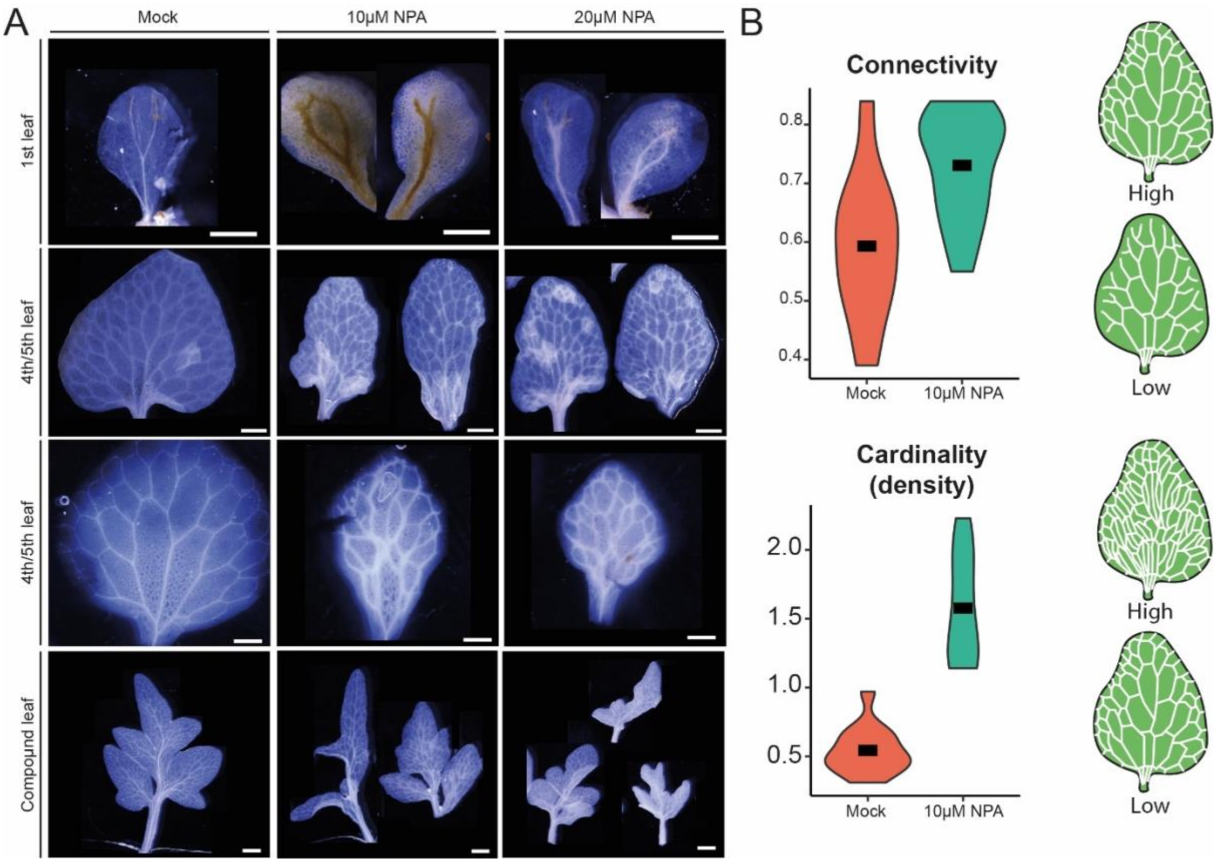

Fig. S4. NPA treatment affects leaf architecture and venation. A) Overview of leaf types and effects of NPA treatment. B) Quantification of venation connectedness (connectivity index; t test p=0.002) and density (cardinality index; t-test p=1.35E-08) of the 4th/5th leaf and examples illustrating observed patterns. Scale bars are 2mm.

Development • Supplementary information

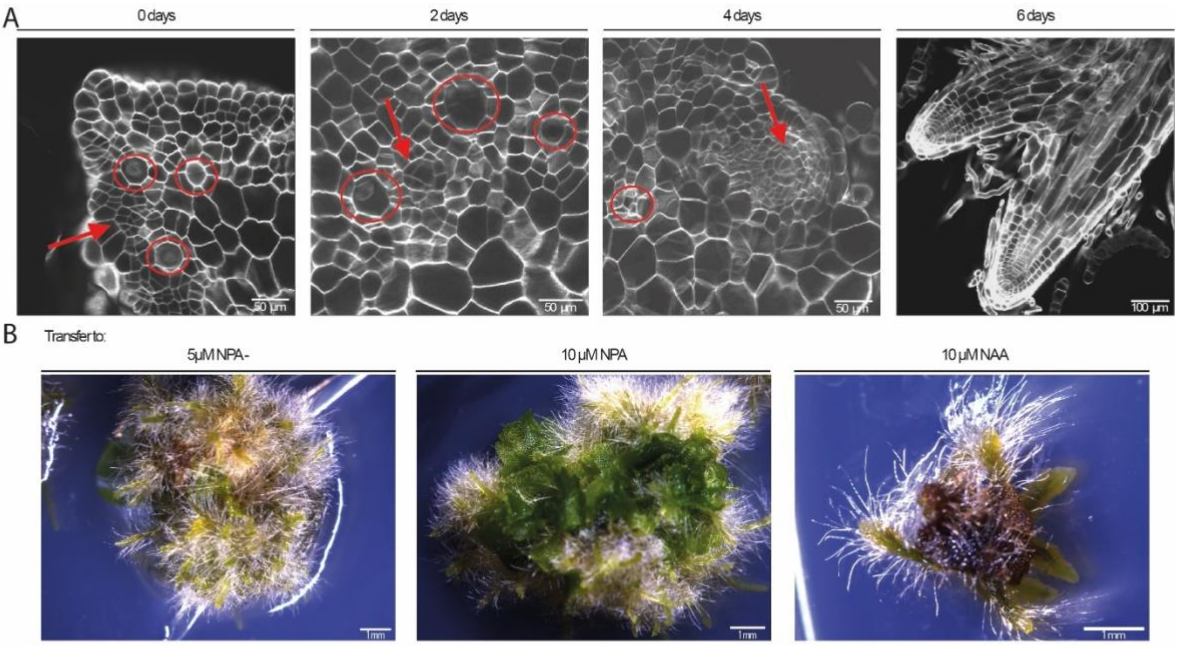

C

Treatment	Days after germination	Frequency ectopic roots
5μM NPA	56 days	27/370
10μM NPA	56 days	1/540
5μM NPA	27 days	0/470
10d 1/2 5μM NPA to ½ MS	35 days	15/42
7d ½MS to 5μM NPA	35 days	11/22
7d ½MS to 10μM NPA	35 days	5/30
7d ½MS to 5μM NAA	35 days	0/32
7d ½MS to 5μM NAA	35 days	29/86

Fig. S5. Characterization of gametophyte-derived root formation. A) Representative pictures of development of ectopic roots on gametophytes collected and cleared from 0 to 6 days after removing plants from media containing 5 μM NPA. Arrows point at abnormal cell morphologies and circles enclose archegonia B) Different hormone treatments lead to the same outcome of the formation of ectopic roots. C) Frequency of gametophytes developing ectopic roots in the different treatments. Scale bars are depicted in the pictures.

Development • Supplementary information

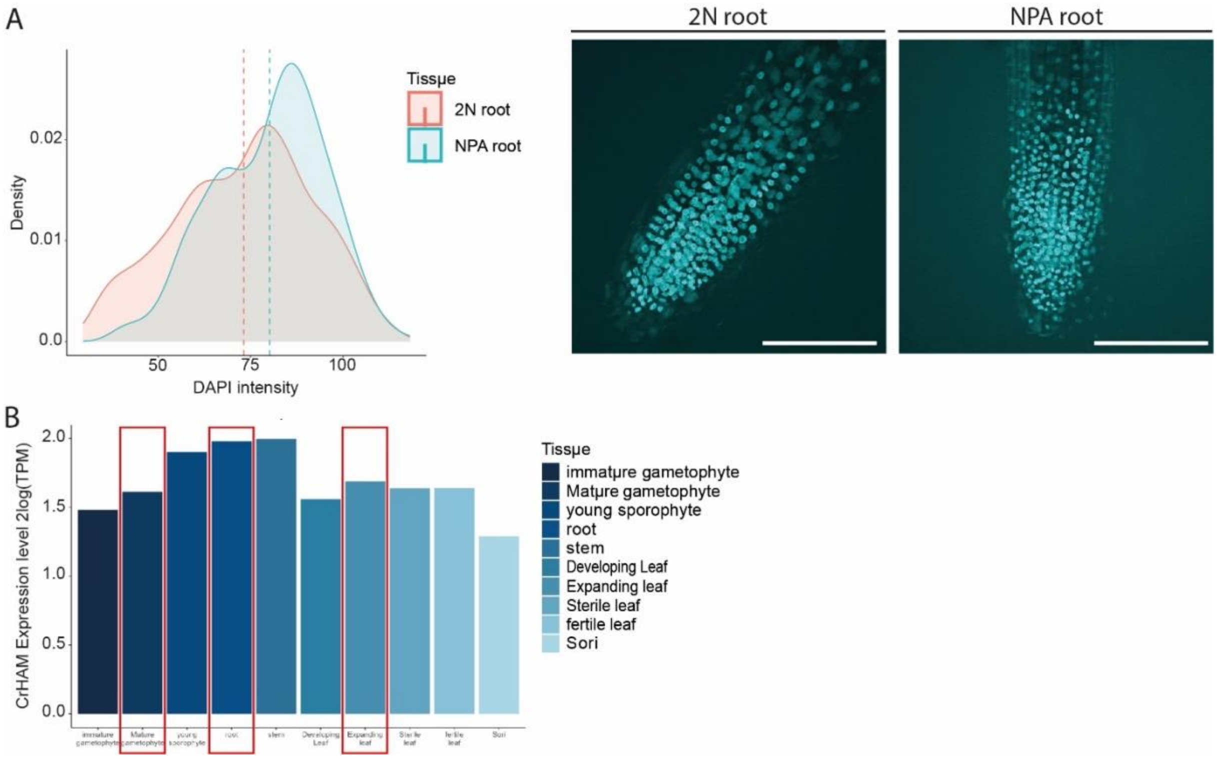

Fig. S6. Ploidy characterization of gametophyte-derived root formation. A) Quantification of nuclear DAPI fluorescence of cleared roots and staining with 50 µg/µL DAPI. B) Expression levels of the *CrHAM* promoter used to drive the H2B-GFP used for ploidy approximation. Scale bars in A are 500 µm.

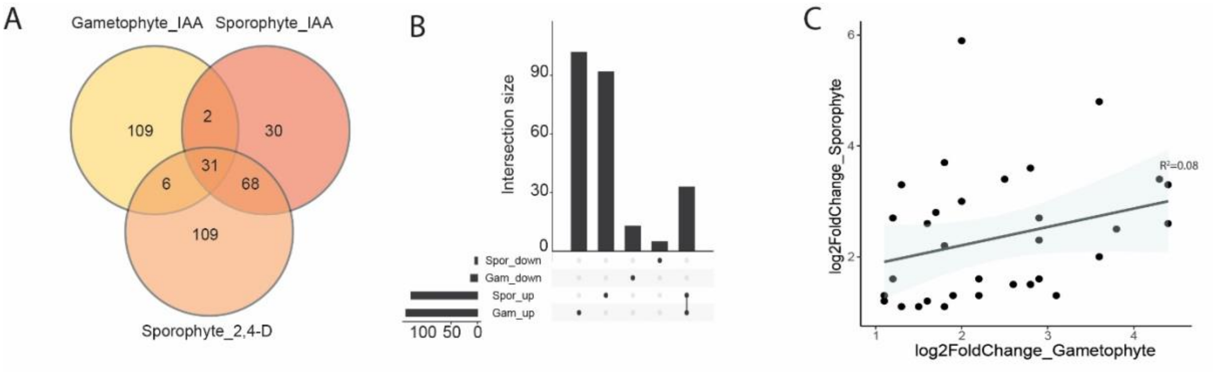

Fig. S7. Overlap between the gametophyte and sporophyte in auxin-responsive genes. A) Overlap between auxin-responsive genes from this study and the prior 2,4-D treatments on sporophytes by Mutte et al. (2018) B) Upset plot showing the overlap between differentially expressed genes at the two life stages. C) Log2FoldChange of the shared 33 DEG between the two different generations.

Development • Supplementary information

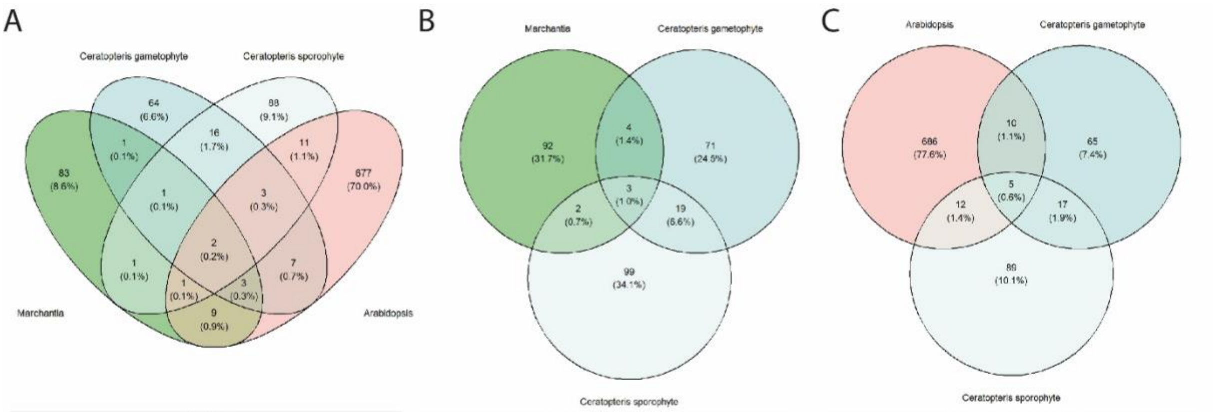

Overlap all

Orthogroup	Arabidopsis ID	Protein family
OG0000023	AT1G20190.1, AT1G26770.2, AT1G69530.3	Expansin
OG0000085	AT1G04100.1, AT1G04240.2, AT1G04250.	Aux/IAA

Overlap Marchantia - Ceratopteris

Orthogroup	Arabidopsis ID	Protein family
OG0001652	AT2G22800.1, AT4G37790.1	homeobox protein HAT9

Overlap Arabidopsis - Ceratopteris

Orthogroup	Arabidopsis ID	Protein family
OG0000106	AT1G23160.1, AT1G28130.1, AT1G48660.2	GH3
OG0000476	AT1G05100.1, AT4G26890.1, AT5G55090.2	MAPKKK
OG0001500	AT1G08290.1, AT1G13290.1, AT1G34790.1	Zinc finger/WIP

Overlap Marchantia - Ceratopteris

Orthogroup	Arabidopsis ID	Protein family
OG0001652	AT2G22800.1, AT4G37790.1	homeobox protein HAT9
OG0000023	AT1G20190.1, AT1G26770.2, AT1G69530.3	Expansin
OG0000085	AT1G04100.1, AT1G04240.2, AT1G04250.	Aux/IAA

Overlap Marchantia - Ceratopteris gametophyte

Orthogroup	Arabidopsis ID	Protein family
OG0000259	AT1G10100.1, AT1G10120.1, AT1G18400.1,	CIB4/BEE1 (bHLH)
OG0000360	AT1G01030.1, AT1G13260.1, AT1G25560.1	RAV TF
OG0000361	AT1G04180.1, AT1G04610.1, AT2G33230.1	YUCCA
OG0000471	AT1G19530.1	RGAT1

Overlap Marchantia - Ceratopteris sporophyte

Orthogroup	Arabidopsis ID	Protein family
OG0011706	Mp1g24230.1, Ceric.01G000300.1.p	Expansin (pollen allergen)
OG0000012	AT1G05240.1, AT1G05250.1, AT1G05260.1,	Peroxidase

Overlap Arabidopsis - Ceratopteris

Orthogroup	Arabidopsis ID	Protein family
OG0000106	AT1G23160.1, AT1G28130.1, AT1G48660.2	GH3
OG0000085	AT1G04100.1, AT1G04240.2, AT1G04250.	Aux/IAA
OG0000023	AT1G20190.1, AT1G26770.2, AT1G69530.3	Expansin
OG0000476	AT1G05100.1, AT4G26890.1, AT5G55090.2	MAPKKK
OG0001500	AT1G08290.1, AT1G13290.1, AT1G34790.1	Zinc finger/WIP

Overlap Arabidopsis - Ceratopteris gametophyte

Orthogroup	Arabidopsis ID	Protein family
OG0001604	AT1G75450.1, AT2G41510.2, AT3G63440.1	CKX (cytokinin oxidase)
OG0000259	AT1G10100.1, AT1G10120.1, AT1G18400.1,	CIB4/BEE1 (bHLH)
OG0000282	AT1G23080.1, AT1G70940.1, AT1G73590.1	PIN
OG0000360	AT1G01030.1, AT1G13260.1, AT1G25560.1	RAV TF
OG0004261	AT1G32270.1, AT2G35460.1, AT2G35980.1	LEA/YLS9
OG0000291	AT1G22900.1, AT1G55210.1, AT1G58170.1	Dirigent
OG0000060	AT1G22640.1, AT1G34670.1, AT1G35515.1	Myb TF
OG0001131	AT3G03170.1, AT3G43850.1, AT4G26288.1,	uncharacterized
OG0000361	AT1G04180.1, AT1G04610.1, AT2G33230.1	YUCCA
OG0000168	AT2G27310.1, AT2G36090.1, AT3G44326.1	F-Box (CFB)

Overlap Arabidopsis - Ceratopteris sporophyte

Orthogroup	Arabidopsis ID	Protein family
OG0000616	AT1G17060.1, AT1G75130.1, AT3G14610.1	Cytochrome
OG0004759	AT2G37380.1, AT2G39370.1	MAKR
OG0003636	AT2G23060.1, AT4G37580.1, AT5G67430.1	HLS1 (N-acetyltransferase
OG0000012	AT1G05240.1, AT1G05250.1, AT1G05260.1	Peroxidase
OG0001688	AT2G23290.1, AT3G50060.1, AT4G37260.1	MYB TF
OG0000548	AT2G22660.2, AT4G37682.1, AT4G37900.1	DUF1399 (glycine-rich domain
OG0008236	AT1G19020.1, AT3G48180.1	uncharacterized transferase
OG0000316	AT1G30210.1, AT1G53230.1, AT1G67260.1	TCP TF
OG0000288	AT1G21460.1, AT1G66770.1, AT3G14770.1,	SWEET
OG0001654	AT2G44740.1, AT5G07450.1, AT5G61650.1	Cyclin P4
OG0000614	AT1G30100.1, AT1G78390.1, AT3G14440.1	NCED
OG0000824	AT1G54095.1, AT1G72510.1, AT1G79770.1	DUF1677

Fig. S8. Orthogroup analysis of auxin-sensitive genes of Marchantia, Ceratopteris and Arabidopsis. A) Overlap between the three species. B) Overlap between Marchantia and the two Ceratopteris generations. The Marchantia dataset is composed of multiple independent datasets ((Kuhn et al., 2024; Mutte et al., 2018)). The Ceratopteris sporophyte dataset is composed of the one described here and an earlier 2.4-D treatments from Mutte et al. (2018) C) Overlap between Arabidopsis and the two Ceratopteris generations.

Development • Supplementary information

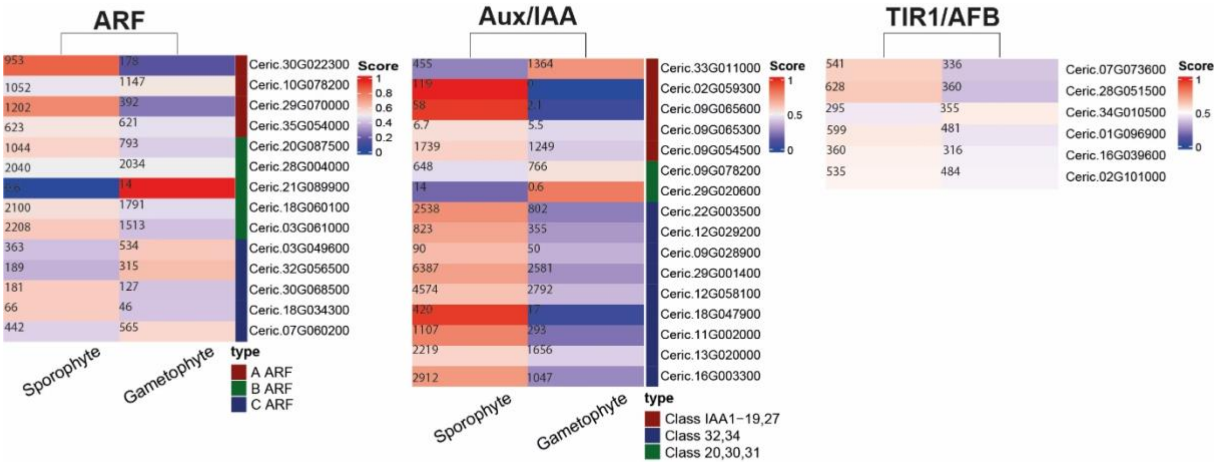

Fig. S9. Heatmaps depicting the relative expression of each NAP gene between the two generations, grouped according to functional/phylogenetic group. Raw expression values are depicted in the boxes (DEseq2 normalized expression). Note that 2 Aux/IAAs showed no expression in the data and are therefore omitted.

Development • Supplementary information

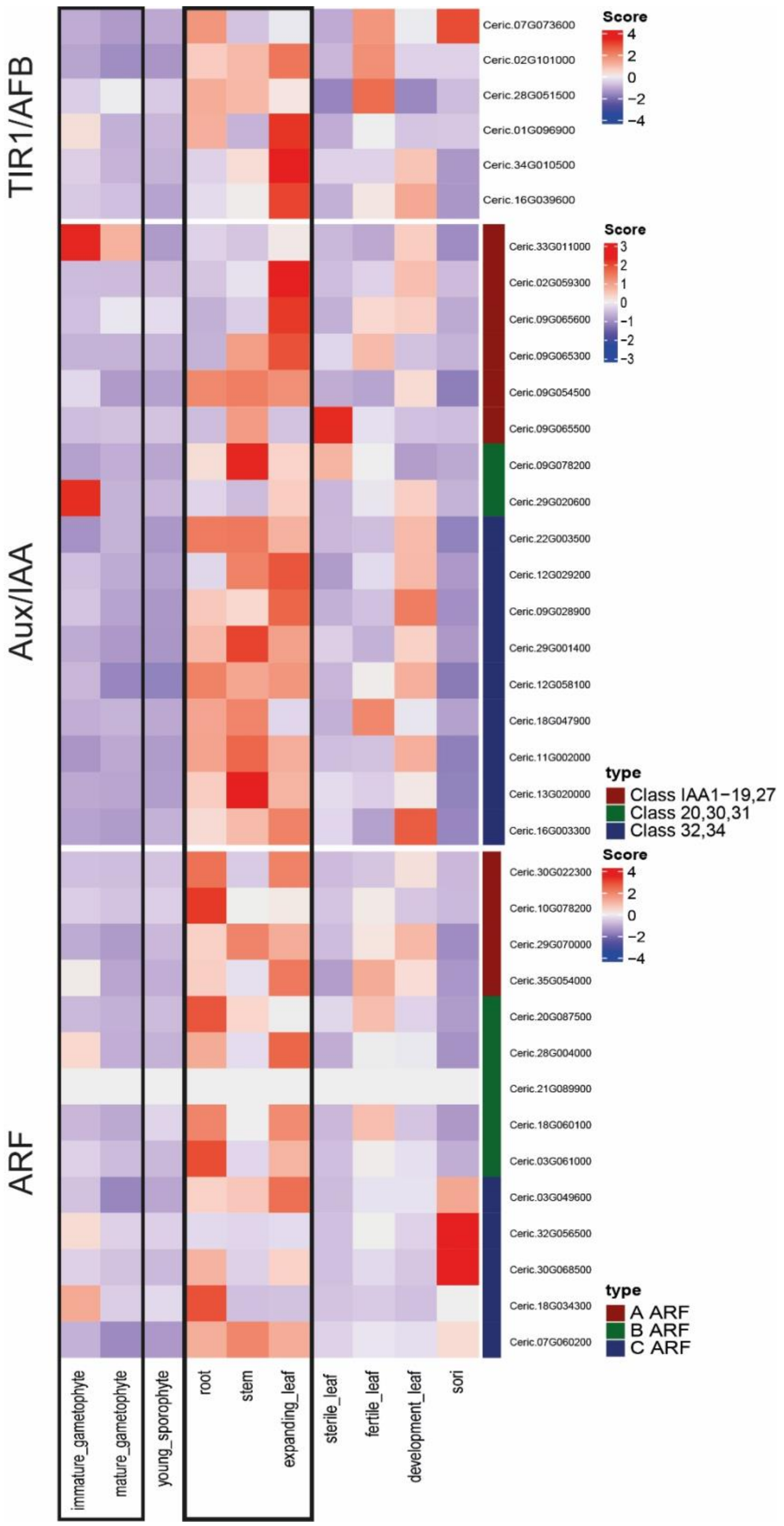

Fig. S10. Heatmaps depicting expression patterns of all NAP genes based on their Z-score. Genes are grouped according to their functional/phylogenetic class. Black boxes emphasize gametophytic tissue and highly expressing sporophytic tissue. Note 1 B-class ARF shows no colour coding due to being to lowly expressed.

Development • Supplementary information

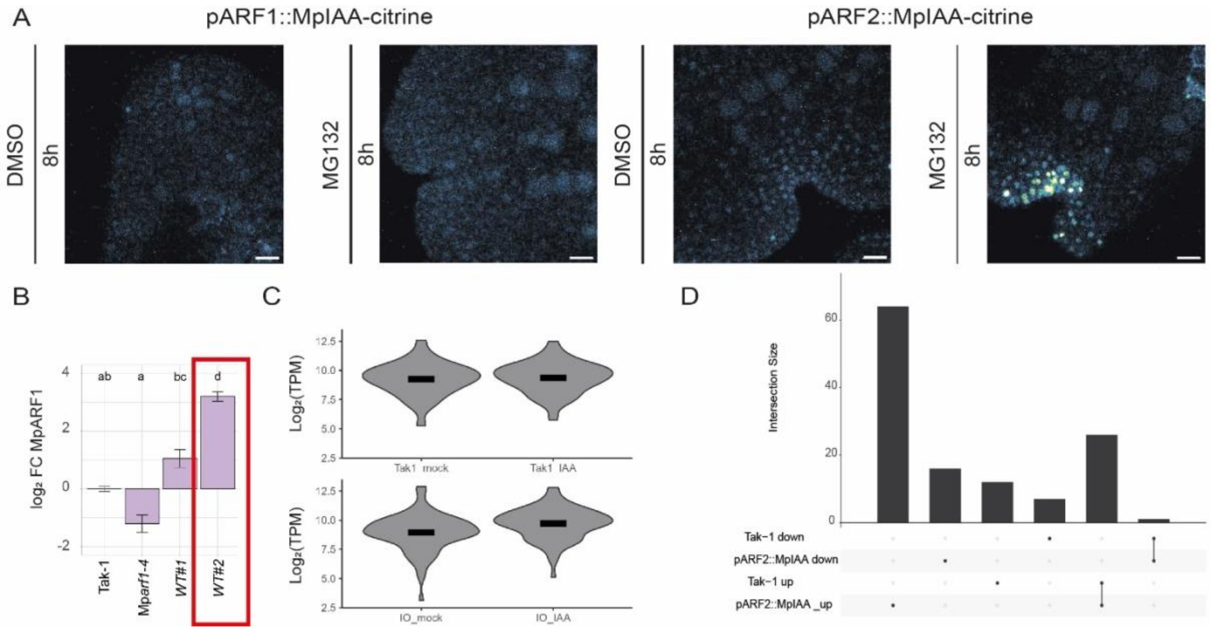

Fig. S11. A) Fluorescence accumulation of MpIAA-Citrine protein in mock-treated gemmae and upon proteosome inhibition (100µM MG132 for 8h). Scale bars are 40 µm. B) qPCR data of arf1-4 mutant complemented MpARF1 showing higher expression levels of ARF1, red box is the lines used in Figure 5. C) Expression values of DEG only present in the pARF2::MpIAA-Citrine (=IO) and not in Tak-1 under mock and IAA conditions. D) upset plot of DEG and their overlap between TAK-1 and pARF2::MpIAA-Citrine.

Development • Supplementary information

Table S1. List of primers used in this study

Name	Description	Sequence
HK120	pARF1 forward (Kato et al., 2020)	CTGCATAAATTGGCTATCATTTATACTACCATG
HK125	pARF1 reverse (Kato et al., 2020)	CCGTCCGAAGATGTGGATGTTG
HK126	pARF2 forward	CGGGGCCAGAGGAGGACTG
HK127	pARF2 reverse	AGAACAGAAGCCAGCGGCA
MpIAA_entry	MpIAA CDS forward (Kato et al., 2015)	CACCTTGGGCACAATGAGTCAAAA
JHG081	MpIAA CDS reverse no stop	TCACACGTTCGGTTGAGTC
HK009	MpARF1 CDS forward (Kato et al., 2020)	CACCATGTATTCTTGTTCGCCG
HK015	MpARF1 CDS reverse (Kato et al., 2020)	GGCGCGCCTCAGGGGCACCCCCGCTGGGCATC
MpACT7_F1	qRT-pPCR – ACTIN 7 forward	AGGCATCTGGTATCCACGAG
MpACT7_R1	qRT-pPCR – ACTIN 7 reverse	ACATGGTCGTTCCTCCAGAC
MpAPT3_F1	qRT-pPCR – ADENINE PHOSPHORIBOSYL TRANSFERASSE 3 forward	CGAAAGCCCAAGAAGCTACC
MpAPT3_R1	qRT-pPCR - ADENINE PHOSPHORIBOSYL TRANSFERASSE 3 reverse	GTACCCCCGGTTGCAATAAG
MpSAND_F1	qRT-pPCR – SAND protein forward	GTTGATGTGTGGCACAAAGG
MpSAND_R1	qRT-pPCR - SAND protein reverse	CAGGCATACGGGAGAAAATG
JR171_MpARF1	qRT-pPCR – MpARF1 forward	CACAAAGATCAGGCTGGCAG
JR172_MpARF1	qRT-pPCR – MpARF1 reverse	ACTTCCGACGGTGACAAGAT

Development • Supplementary information
