# Supplementary material for: Analysis of auxin responses in the fern Ceratopteris richardii identifies the developmental phase as a major determinant for response properties
Source: Development. 2024 Sep 26;151(20):dev203026. doi: 10.1242/dev.203026 (PMC11449451; doi:10.1242/dev.203026)
Supplement: Supplementary file 1 [file develop-151-203026_review_history.pdf]

Analysis of auxin responses in the fern *Ceratopteris richardii* identifies developmental phase as a major determinant for response properties

Sjoerd Woudenberg, Melissa Dipp Alvarez, Juriaan Rienstra, Victor Levitsky, Victoria Mironova, Enrico Scarpella, Andre Kuhn and Dolf Weijers
DOI: 10.1242/dev.203026

Editor: Liam Dolan

Review timeline

Original submission:	3 May 2024
Editorial decision:	27 June 2024
First revision received:	15 August 2024
Accepted:	16 August 2024

Original submission

First decision letter

MS ID#: DEVELOP/2024/203026

MS TITLE: Analysis of auxin responses in the fern Ceratopteris richardii identifies tissue ontogeny as a major determinant for response properties

AUTHORS: Sjoerd Woudenberg, Melissa Dipp Alvarez, Juriaan Rienstra, Victor Levitsky, Victoria Mironova, Enrico Scarpella, Andre Kuhn, and Dolf Weijers

I have now received all the referees reports on the above manuscript, and have reached a decision. The referees' comments are appended below, or you can access them online: please go to BenchPress and click on the 'Manuscripts with Decisions' queue in the Author Area.

The overall evaluation is positive and we would like to publish a revised manuscript in Development, provided that the referees' comments can be satisfactorily addressed. Please attend to all of the reviewers' comments in your revised manuscript and detail them in your point-by-point response. If you do not agree with any of their criticisms or suggestions explain clearly why this is so. If it would be helpful, you are welcome to contact us to discuss your revision in greater detail. Please send us a point-by-point response indicating your plans for addressing the referees' comments, and we will look over this and provide further guidance.

Reviewer 1

Advance summary and potential significance to field

Analysis of auxin responses in the fern Ceratopteris richardii identifies tissue ontogeny as a major determinant for response properties Woudenberg et al

In this paper, the authors tease apart whether auxin responses are generation-dependent (ontogeny) or determined by lineage/ancestry (phylogeny). They use Ceratopteris as a model system, as it has free-living haploid and diploid generations, and assess differences with previously reported auxin responses in liverwort gametophytes and Arabidopsis sporophytes. However, a key

© 2024. Published by The Company of Biologists under the terms of the Creative Commons Attribution License (https://creativecommons.org/licenses/by/4.0/).

auxin-treated tissue missing from these analyses is the bryophyte sporophyte. As an overall comment, the conclusions drawn from analysing auxin responses in bryophytes vs tracheophytes (for gametophytes) and amongst tracheophytes (for sporophytes) can be more clearly stated in the paper: In the former case, one can infer the nature of the ancestral land plant gametophyte, while in the latter case one can only infer the nature of the sporophyte in the euphyllophyte ancestor. These comparisons are not symmetrical and I feel the paper is very confidently asserting that ontogeny is the major determinant of auxin responses in plants when we don't really view the entirety, even with the significant contributions made by this paper. Below are a list of specific suggestions or criticisms that could improve the paper.

Comments for the author

A first finding of the paper includes the common formation of rhizoid cell identity in fern and liverwort gametophytes (also likely in moss, hornwort, and lycophytes). This result suggests that ancestral land plant gametophyte produced rhizoids in response to auxin and was likely free living and autotrophic. This could be specified more clearly in the discussion. One very interesting result that is not addressed in the paper is that Marchantia has higher sensitivity to auxin analogues (NAA) than Ceratopteris in the gametophytic generation.
For example, in Figure 1E (1uM NAA) it seems much of Marchantia produces rhizoids, while in Ceratopteris growth inhibition by the same NAA concentration is not as pronounced. Could the authors include a normalized curve of auxin response for the Marchantia gametophyte, this would show whether there are lineage-specific responses to exogenous auxin even in the gametophytic generation. Is there a higher robustness to hormonal perturbations in fern gametophytes compared to bryophytes and is this due to genetic redundancy of the auxin signalling machinery in ferns? — in this regard, something that was not obvious in the paper: how many genes for components of nuclear auxin response exist in Ceratopteris, and what proportion are expressed in the different generations?

Secondly, the paper also shows how auxin promotes root branching in fern sporophytes as well as root hair cell (?) expansion. This has parallels with angiosperm auxin-responses in the sporophyte. Finally, auxin transport has a role in leaf vasculature formation in ferns just as it happens in seed plant leaves. The paper misses to explain that even though leaves evolved independently in euphylophytes, both organs evolved as modifications of the shoot. If the shoot is homologous amongst tracheophytes, then we expect shared cell identity specification (i.e vasculature) by auxin transport in two (independently) modified shoots. This could be addressed in line 422 in the discussion where the authors point at the intriguing convergence of leaf programs between ferns and seed plants.

Thirdly, gametophytes treated with auxin transport antagonist NPA and later moved to mock media, produced a root-like program that included vasculature formation and a root apical cell as an example of 'partial' apogamy. The ploidy of gametophytic rootlets was corroborated by GFP-density assays using a transgenic line with ubiquitous expression in diploid and haploid tissue. However, I am not sure whether this is sensu stricto apogamy as a whole sporophyte-like tissue (including roots and shoots) was not form after NPA application. These results could simply show that auxin perturbation can induce the root program, but perhaps not the sporophytic program. In mosses apogamy is obtained by interfering with the polycomb repressive complex and the discussion should address whether auxin transport has a role antagonising polycomb or perhaps simply the root program. To me it is not clear whether auxin maxima or minima is triggering this effect. Could the authors perform a similar experiment (media transfer) with auxin biosynthesis antagonists such as PPBo or yucassin to address this question?

Regarding amplitude in sporophytic responses, why are only the top 20 genes selected to assess differences in logFC amplitude in mock vs iaa-treated plants? Is this arbitrary? In figure 4B, it does seem like the average FC in auxin-treated/mock sporophytes is higher than in gametophytes, but the authors do not suggest that the distribution in sporophytes seems bimodal? If this is the case, sporophyte expressed genes in Ceratopteris are responding to auxin in two modes, one set of genes shows a gametophytic-like response and a second set of genes shows higher or more intense responses to auxin. Could the authors show whether the latter group consists of genes only expressed in the sporophyte? How many genes are shared in both auxin response between generations? Definitely a statistical test on bimodal vs normal distribution could be worth doing.

© 2024. Published by The Company of Biologists under the terms of the Creative Commons Attribution License (https://creativecommons.org/licenses/by/4.0/).

As mentioned in the first part of the review, missing in the discussion is future comparisons with auxin-treated bryophyte sporophytes and what sort of information could be obtained from these. For example whether the proposed high gene activation amplitude observed in auxin-treated sporophytes is ancestral to plants or occurred within the tracheophyte lineage. Or whether the sporophyte-enriched auxin response element-like motifs date back to the land plant or tracheophyte ancestor?

A few specific comments:

Lines 71-73: Is gravity sensing really different between lineages? In liverworts tht form negatively gravitropic gametangiophores, typical statoliths have been documented and PIN mediated auxin transport is required for the gravitropic response, shifting auxin away from basipetal transport.

Line 81, in ferns (and lycophytes) both … generations are autotrophic. Gametophytes in heterosporous lycophytes are endosporic and therefore may not be autotrophic.

Line 166. 'The tissues that show responses to auxin in the Ceratopteris sporophyte and gametophyte are not homologous, except for root hairs, which are transcriptionally homologous to rhizoids (Jones and Dolan 2012; Menand et al., 2007).' — The term 'transcriptional homology' is misleading as it suggests root hairs and rhizoids are morphologically homologous. Homology really only means descended from a common ancestor.
The scenario where two morphologically similar but unrelated structures are specified by the same transcription factors, is just as well explained by co-option. Rhizoids and root hairs are specified by orthologous genes in non-homologous tissues.

Line 226, "Interestingly, this root outgrowth only seems to happen upon transferring media and takes time…"
Consider revising this sentence. For example: "Interestingly, gametophytic root outgrowth only happens ~50 days after a media transfer event, explaining why this response…"

Lines 283 – 285 gene counts units (TPM) are missing across all sentences, for example in (log2 = 2.76 ?!).
Please add TPM after each log2, this should be done for Figure 4E where y-axis has no units.

Reviewer 2

Advance summary and potential significance to field

Here mi review and comments on the paper by Woudenberg et al, titled
¨Analysis of auxin responses in the fern Ceratopteris richardii identifies tissue ontogeny as a major determinant for response properties¨
Authors contribute with a vast collection of experiments to study the similarities and differences among the responses of auxin between the Ceratopteris gametophyte versus Marchantia thalli and those among the Ceratopteris sporophyte versus Arabidopsis roots. Authors use phenotypic analyses and transcriptomic and proteomic approaches to explore such responses to exogenous auxin applications and auxin transport inhibition.
Authors claim that their data led them to conclude that the responses to auxin homeostasis perturbations are determined by tissue ontogeny.

While the study and data can be of interest to the Evo-Devo field, there are major concerns on the paper that I consider authors should solve to accept the publication of this manuscript in Development. Please find my reasons below.

Comments for the author

I consider that the manuscript in its current status is not suitable for publication in Development, however I encourage authors to carefuully read the following comments and suggestions in order to resubmit and improved version on the manuscript.

© 2024. Published by The Company of Biologists under the terms of the Creative Commons Attribution License (https://creativecommons.org/licenses/by/4.0/).

Major comments based on the most important statements and conclusions along the manuscript.

1. ¨We find that phenotypically, Ceratopteris gametophytes respond similar to Marchantia gametophyte.¨
Results related to Figure 1 are not fully clear, although growth inhibition of Both Marchantia and Ceratopteris is evident, as previously reported in other studies, there is no evidence of the claimed tissue ontogeny neither in response to exogenous auxin nor to auxin transport inhibition, except for the effect on rhizoid ectopic formation and elongation, a phenotype previously reported for both Marchantia and Ceratopteris gametophytes.
The disorganization of Ceratopteris lateral meristem caused by exogenous auxin or NPA may be a novel phenotype that strengthens the claimed effect on similar tissues like stem cell niches, unfortunately, this is not compared with the effects of auxin or NPA on Marchantia apical notches.
From all results described and related to Figure 1, only the effects on rhizoids is robust, as well as the growth inhibition effects, however, this has been previously reported.
It would be of great value for the paper to include, side by side, the changes cause by exogenous auxin and NPA in Ceratopteris lateral meristems compared to those in the apical notches of Marchantia young thalli or gemmae.

2. ¨Ceratopteris sporophytes closely resemble responses of Arabidopsis sporophytes¨.
This response was partially demonstrated 20 years ago by Hou etal (2004). Unfortunately, the description of the results shown in Figure 2 is ambiguous as an example the following paragraph: ¨This increased branching was pronounced after 12 days of growth in auxin supplemented medium (Fig. 2C,E), but an initial response was already visible after three days (Fig. S2A,B). Interestingly, the increased branching was not visible in the first appearing root of the young sporophytes, clearly showing that branching is root age/stage-dependent, and potentially explaining why this response is sometimes not visible¨.

These results are poorly analyzed and described, one would expect that authors would describe and emphasize the novel findings compared to Hou et al 2004, since Hou and coworkers already showed the effect of diverse auxinic molecules over the fifth root of Ceratopteris with great detail. They already showed that the response of the root to exogenous auxin is stage-dependent.
Indeed in the discussion section, the authors claim: ¨In summary, by studying the fern Ceratopteris richardii, a missing link in our understanding of auxin biology has been filled¨ this implies that their work for the first time fills the gap, when Hou et al did it for the sporophyte in 2004 and Withers metal, for the gametophyte in 2023. Instead of such a statement, one would expect that authors could discuss how their work coincides, or not, with previous studies, and integrate the findings of the three studies. Once their results are well described.
Another major observation is the description of the analyses of exogenous auxin on Arabidopsis root hair development versus those of Ceratopteris in the roots of the sporophyte. What are the conclusions, similarities, or differences between rhizoids and root hairs?

3 ¨Disrupting auxin transport can induce a sporophytic program¨
This could be a major finding of this study, however, it is not presented in a detailed manner to avoid a wrong conclusion. Authors claim that inhibiting the transport of auxin causes a major reprogramming of the gametophyte to a sporophyte and they sustain this by the observation of root formation after prolonged treatment with NPA. However to sustain that NPA induces the formation of ectopic roots from tissues of non-fertilized hermaphrodite gametophytes, a time-lapse of the process should be registered. How can authors discard that during the prolonged treatment the self-fertilization of the hermaphrodite gametophyte occurred and the root emergence is only accelerated? Authors show a single picture of putative root apical cell, how often this occurs in NPA treatment versus non-treated gametophytes? This never occurred in non-treated gametophytes cultured during the same, prolonged, time?. Are these claimed ¨transdifferentiation¨ events occurring also in the male gametophyte or only in the hermaphrodite?
A time-lapse observation versus the control recorded and in a system in which during the prolonged culture could not cause self-fertilization could strengthen and make more robust this interesting observation.

Finally, the most novel data generated by this study is the comparative transcriptomic profiling, in response to exogenous auxin, among Marchantia, Ceratopteris, and Arabidopsis. These analyses lead to the identification of up-regulated genes among which, as expected, are genes related to

© 2024. Published by The Company of Biologists under the terms of the Creative Commons Attribution License (https://creativecommons.org/licenses/by/4.0/).

auxin-synthesis, transport, and signaling. Unfortunately, the bulk transcriptomic analyses do not allow the identification of cell-type specific genes that supports the hypothesis of a conserved response to auxin at cell type level. My major concern is again the final interpretation of the data which is too vague, and example of this is the following paragraph: ¨The transcriptional nature of the response clearly depends on the ontogeny of the organism and vice-versa shapes its development due to their interdependency¨.

4. ¨… we find that auxin may control the alternation of generations.¨
The phosphor-proteomics results are also quite different among species, but authors do not explain clearly why. Did they compare phosphor-proteomics from Marchantia thalli versus that of Ceratopteris hermaphrodite gametophyte? Yes or not and why not? Did they compare Arabidopsis roots versus Ceratopteris roots phosphor-proteins, Yes or not and why not? If the answer is not, how can authors and readers could conclude some reasonable comparison or claim ontogeny conservation? Could they elaborate more in the discussion?

Minor comments:

Figure 1 legend, should be specific when referring to Marchantia or Ceratopteris, non-expert readers will be confused when authors are describing cells from one or the other. Some imagesin in Figure 1A, D and E are so small that it is difficult to observe what the authors claim, besides growth reduction. What are red asterisks indicating in images in Figure 1 Panel F?
There are several typos along the text, please revise again.

Reviewer 3

Advance summary and potential significance to field

The study compares the effects of auxin on the development of the gametophyte phase and sporophyte phase of the Ceratopteris richardii life cycle and then compares this to what is known in the bryophyte Marchantia polymorpha, and the angiosperm, Arabidopsis thaliana. This is important as ferns are an ideal system of comparison as they have free living gametophytes and sporophytes and comparisons between Marchantia and Arabidopsis were not comparing the same phase of the life cycle. This study will lay the framework for understanding the role of auxin in the evolution and development of the land plant body plan.

Comments for the author

I would suggest the use 'phase' as opposed to 'ontogeny'. The gametophyte ontogeny of bryophytes, ferns and angiosperms differ. Same for sporophyte ontogeny. But here they are comparing gametophyte phases or sporophyte phases the result of meiosis or syngamy respectively.
Although not inaccurate, I think it is clearer to use ‘phase’ instead of ‘generation’. As both phases are needed to move from one generation to the next (unless apospory or apogamy occurs)
Line 81 - change to ‘Some lycophytes’. The selaginellales and isoetales are heterosporous and endosporic - that is the gametophytes develop inside the spore while sometimes still attached to the sporophyte. So the phases are not always independent. These gametophytes also lack chlorophyll. Some lycopodiales gametophytes are colorless and buried underground.

Line 94 - there are many experiments done with auxin in fern species such as Wetmore & Pratt, 1949; Voeller 1960; Briggs & Steve, 1959; Steve & Briggs, 1960; Peterson and Cutter 1969a,b ; Reviewed in White 1971 Botanical Review 37: 509-540. these should be included as relevant.

Line 101 - how do responses in physcomitrium gametophytes compare?

Line 125 - typo change to ‘importantly’

Lines 132-135 - auxin has an affect on the egg cell of Arabidopsis. How far did you follow ontogeny of the ceratopteris archegonium? It is not possible to see the egg cell in supplemental Fig S1b. Also, did fertilization occur? Were there any impacts on embryo ontogeny? Auxin has a strong impact on embryo patterning in angiosperms.`21

© 2024. Published by The Company of Biologists under the terms of the Creative Commons Attribution License (https://creativecommons.org/licenses/by/4.0/).

Lines 134–5 - are there any auxin studies in physcomitrium?

Line 136 - what are the red stars in panel F of fig 1?

Lines 173-4 - laminar morphology of a gametophyte is not similar to euphyllophyte leaves

Line 175 - the evolution of leaves is far from settled. It is thought that leaves evolved once, twice (in lycophytes and again euphyllophytes (ferns + seed plants), or up to 12 times (mostly in the ferns). All data needs to be considered, fossil, morphological, and molecular. This is debate is far from settled and this sentence should reflect that.

Line 186 - were there any auxin affects on timing of sporangia development or position of sporangia or morphology of sporangia?

Line 207 - where did these sporophyte roots arise from? Randomly? Near the notch meristem? On the rhizoid side of the gametophyte?

Line 322 - why not knock these down Ceratopteris to directly assess the effect on duplications in aux/Iaa or a-type arf families?

Lines 422-4 - see comment on leaf evolution above. Also auxin experiments on ferns in another comment above.

Lines 455 - typo change to 'advancements'

Lines 455-7 - knockdowns are possible in ceratopteris - see bui et al, youngstrom et al, withers et al and placket et al. Along with recent reports of crispr in ceratopteris.

First revision

Author response to reviewers' comments

Response to reviewers

We are grateful to all three reviewers for their thoughtful and critical assessment of our manuscript, and for raising concerns and flagging inconsistencies and points for clarification. We have considered all points raised and have added new data and made pertinent revisions to the text. We believe that this makes for a better, more balanced version of our manuscript. Below, we respond to each point individually.

Reviewer 1:

1. A first finding of the paper includes the common formation of rhizoid cell identity in fern and liverwort gametophytes (also likely in moss, hornwort, and lycophytes). This result suggests that ancestral land plant gametophyte produced rhizoids in response to auxin and was likely free living and autotrophic. This could be specified more clearly in the discussion. One very interesting result that is not addressed in the paper is that Marchantia has higher sensitivity to auxin analogues (NAA) than Ceratopteris in the gametophytic generation. For example, in Figure 1E (1uM NAA) it seems much of Marchantia produces rhizoids, while in Ceratopteris growth inhibition by the same NAA concentration is not as pronounced. Could the authors include a normalized curve of auxin response for the Marchantia gametophyte, this would show whether there are lineage- specific responses to exogenous auxin even in the gametophytic generation. Is there a higher robustness to hormonal perturbations in fern gametophytes compared to bryophytes and is this due to genetic redundancy of the auxin signalling machinery in ferns? — in this regard, something that was not obvious in the paper:

© 2024. Published by The Company of Biologists under the terms of the Creative Commons Attribution License (https://creativecommons.org/licenses/by/4.0/).

how many genes for components of nuclear auxin response exist in Ceratopteris, and what proportion are expressed in the different generations?

We thank the reviewer for this comment. We have included a growth curve of *Marchantia* thallus to exogenous applied auxins to allow for a better comparison. We see a similar response and also sensitivity - the same NAA concentrations cause a clear growth reduction in both species (1 µM). For IAA, it is less clear although both plants show a clear effect at 1 µM. This suggests that sensitivity is comparable for long-term growth experiments. However, it should be noted that in Ceratopteris, growth started from a single-celled spore, while in Marchantia, growth started from a multi-cellular gemma.

We do not find a clear correlation between the number of components in the nuclear auxin pathway and the sensitivity of the species, as *Marchantia* only has 3 ARFs, while Ceratopteris has 14 ARF (supplemental figures S6/S7). We added these details also in the text.

2. Secondly, the paper also shows how auxin promotes root branching in fern sporophytes as well as root hair cell (?) expansion. This has parallels with angiosperm auxin-responses in the sporophyte. Finally, auxin transport has a role in leaf vasculature formation in ferns just as it happens in seed plant leaves. The paper misses to explain that even though leaves evolved independently in euphylophytes, both organs evolved as modifications of the shoot. If the shoot is homologous amongst tracheophytes, then we expect shared cell identity specification (i.e vasculature) by auxin transport in two (independently) modified shoots. This could be addressed in line 422 in the discussion where the authors point at the intriguing convergence of leaf programs between ferns and seed plants.

We thank Reviewer 1 for the opportunity to clarify that important point. We agree with the reviewer that the shoot may be homologous among vascular plants. And if leaves started out as vascularized shoot branches, the major veins that derive from those vascularizations would also be homologous among vascular plants. However, the lamina that connects those shoot branches and the minor veins that connect those major veins evolved independently in ferns and flowering plants (e.g., Doyle & Hickey, 1976; Paliwal et al., 1976; Boyce & Knoll, 2002; Galtier, 2010; Sanders et al., 2007). Accordingly, cells at early stages of vein development in ferns and flowering plants have different histochemical properties, and vein formation initiates, proceeds, and terminates differently in those plant groups (e.g., Pray, 1955; Pray, 1960). Moreover, closed vein loops, which result from bilateral connection of minor veins to other veins, also evolved independently in ferns and flowering plants (e.g., Doyle & Hickey, 1976; Paliwal et al., 1976; Boyce & Knoll, 2002). Therefore, given the independent evolution of minor veins and closed loops in both ferns and flowering plants, we think it's unexpected that the number of veins and their connectedness depend on polar auxin transport in both those plant groups. We've included those considerations in the revised manuscript.

3. Thirdly, gametophytes treated with auxin transport antagonist NPA and later moved to mock media, produced a root-like program that included vasculature formation and a root apical cell as an example of 'partial' apogamy. The ploidy of gametophytic rootlets was corroborated by GFP-density assays using a transgenic line with ubiquitous expression in diploid and haploid tissue. However, I am not sure whether this is sensu stricto apogamy as a whole sporophyte-like tissue (including roots and shoots) was not form after NPA application. These results could simply show that auxin perturbation can induce the root program, but perhaps not the sporophytic program. In mosses apogamy is obtained by interfering with the polycomb repressive complex and the discussion should address whether auxin transport has a role antagonising polycomb or perhaps simply the root program. To me it is not clear whether auxin maxima or minima is triggering this effect. Could the authors perform a similar experiment (media transfer) with auxin biosynthesis antagonists such as PPBo or yucassin to address this question?

We thank the reviewer for these good suggestions. We added a section in the discussion addressing the role of polycomb and epigenetic reprogramming for the ectopic root formation, but it is very much an open question if auxin has a role antagonising polycomb activity.

To our understanding, auxin maxima play an important role in triggering the ectopic roots,

© 2024. Published by The Company of Biologists under the terms of the Creative Commons Attribution License (https://creativecommons.org/licenses/by/4.0/).

because we see the same effect with NAA treatments (Fig. S5B). We added this point to the discussion. Additionally grew gametophytes on the signaling antagonist PEO-IAA (TIR1- inhibitor), or the biosynthesis inhibitors L-Kynurenine (L-Kyn) together with Yucasin and L- Kyn together with Yucasin DF. For the latter, the binding pockets are conserved between Marchantia and *Arabidopsis*, end we expect them to work in Ceratopteris. However, to see these ectopic root formations we need to grow plants over a long period, and we did not observe any growth defects over such time scales. For PEO-IAA, we mainly see a reduction or arrest of growth. Additionally, growth on L-Kyn of Ceratopteris gametophytes is already reported and shows a very different morphology than the here reported NPA-treated plants (Withers et al. (2023). Together this suggest indeed that auxin maxima play a role.

4. Regarding amplitude in sporophytic responses, why are only the top 20 genes selected to assess differences in logFC amplitude in mock vs iaa-treated plants? Is this arbitrary? In figure 4B, it does seem like the average FC in auxin-treated/mock sporophytes is higher than in gametophytes, but the authors do not suggest that the distribution in sporophytes seems bimodal? If this is the case, sporophyte expressed genes in Ceratopteris are responding to auxin in two modes, one set of genes shows a gametophytic-like response and a second set of genes shows higher or more intense responses to auxin. Could the authors show whether the latter group consists of genes only expressed in the sporophyte?
How many genes are shared in both auxin response between generations? Definitely a statistical test on bimodal vs normal distribution could be worth doing.

We thank the reviewer for this suggestion and interesting point, we address all the points in reverse order. The number of responsive genes is visible in figure S5A. In total they share 33 DEG between the two generations, which is about 1/3 of the total amount of DEG in the sporophyte. We included a plot of only the shared DEG and their amplitude in the supplemental Figure 5C. This shows that there is no clear correlations, therefore we added a line in the manuscript stating: “The shared DEG show no clear distinction in their amplitude between the two different generations (Fig. S5C), suggesting that this difference in amplitude is partly due to the generation-specific targets.”.

Following the reviewer’s suggestion, we tried multiple statistical tests for bimodality (dipp test, excess mass test, critical bandwidth test) and all of these are non-significant, suggesting that there is not enough evidence to consider it bimodal.

We only selected the 20 highest and lowest responsive genes to visualize the difference in having a low versus high response. This is completely arbitrary, but it shows the same picture if you select the top 30 and bottom 30, which would comprise already 50% of all sporophytic DEG. The top 20 is also based on Mutte et al. (2018) as we did a similar approach in that paper.

5. As mentioned in the first part of the review, missing in the discussion is future comparisons with auxin- treated bryophyte sporophytes and what sort of information could be obtained from these. For example, whether the proposed high gene activation amplitude observed in auxin-treated sporophytes is ancestral to plants or occurred within the tracheophyte lineage. Or whether the sporophyte-enriched auxin response element-like motifs date back to the land plant or tracheophyte ancestor?

We thank the reviewer for this interesting point, we agree this would be a very interesting future endeavour. We added a paragraph about this to the discussion.

6. Lines 71-73: Is gravity sensing really different between lineages? In liverworts tht form negatively gravitropic gametangiophores, typical statoliths have been documented and PIN mediated auxin transport is required for the gravitropic response, shifting auxin away from basipetal transport.

We adjusted these lines, to make our point more clear.

7. Line 81, in ferns (and lycophytes) both … generations are autotrophic. Gametophytes in heterosporous lycophytes are endosporic and therefore may not be autotrophic.

© 2024. Published by The Company of Biologists under the terms of the Creative Commons Attribution License (https://creativecommons.org/licenses/by/4.0/).

Removed the Lycophytes statement, as their development is indeed completely different.

> *8. Line 166. 'The tissues that show responses to auxin in the Ceratopteris sporophyte and gametophyte are not homologous, except for root hairs, which are transcriptionally homologous to rhizoids (Jones and Dolan, 2012; Menand et al., 2007).' — The term 'transcriptional homology' is misleading as it suggests root hairs and rhizoids are morphologically homologous. Homology really only means descended from a common ancestor. The scenario where two morphologically similar but unrelated structures are specified by the same transcription factors, is just as well explained by co-option. Rhizoids and root hairs are specified by orthologous genes in non-homologous tissues.*

Adjusted this on 2 places in the text.

> *9. Line 226, "Interestingly, this root outgrowth only seems to happen upon transferring media and takes time…" Consider revising this sentence. For example: "Interestingly, gametophytic root outgrowth only happens ~50 days after a media transfer event, explaining why this response…"*

Adjuste
d

> *10. Lines 283 – 285 gene counts units (TPM) are missing across all sentences, for example in (log2 = 2.76 ?!). Please add TPM after each log2, this should be done for Figure 4E where y-axis has no units.*

We added and changed this. Note that it was not TPM but DEseq2 normalized expression values (median of ratio's).

Reviewer 2:

> *11.¨We find that phenotypically, Ceratopteris gametophytes respond similar to Marchantia gametophyte.¨ Results related to Figure 1 are not fully clear, although growth inhibition of Both Marchantia and Ceratopteris is evident, as previously reported in other studies, there is no evidence of the claimed tissue ontogeny neither in response to exogenous auxin nor to auxin transport inhibition, except for the effect on rhizoid ectopic formation and elongation, a phenotype previously reported for both Marchantia and Ceratopteris gametophytes. The disorganization of Ceratopteris lateral meristem caused by exogenous auxin or NPA may be a novel phenotype that strengthens the claimed effect on similar tissues like stem cell niches, unfortunately, this is not compared with the effects of auxin or NPA on Marchantia apical notches. From all results described and related to Figure 1, only the effects on rhizoids is robust, as well as the growth inhibition effects, however, this has been previously reported. It would be of great value for the paper to include, side by side, the changes cause by exogenous auxin and NPA in Ceratopteris lateral meristems compared to those in the apical notches of Marchantia young thalli or gemmae.*

We thank the reviewer for these good suggestions. We grew *Marchantia* gemma and young thalli under exogenous auxin and NPA treatments similar to Ceratopteris at different concentrations. However, we see no clear phenotypic response. The apical notches in *Marchantia* just disappear upon treatment and differentiate to rhizoids. This is very much in line with other literature that reports the induction of rhizoids and growth reduction in *Marchantia* upon auxin treatments (Kato et al. 2015), or a smaller lobe between apical notches (Flores-Sandoval et al. 2015). Other auxin phenotypes described in *Marchantia* (like air chambers, archegoniophores) can not be compared to Ceratopteris as such tissues are missing. The smaller lobe between apical notches is also in line with the role of auxin minima for notch formations. We added an extra supplemental figure to show the response in *Marchantia* gemmae grown for 3 and 5 days on NAA/NPA-supplemented media (Fig. S2).

Additionally, the lateral meristem in Ceratopteris is completely different from the apical notches in

© 2024. Published by The Company of Biologists under the terms of the Creative Commons Attribution License (https://creativecommons.org/licenses/by/4.0/).

Marchantia. For instance, the *Marchantia* apical notch depends on a singular apical cell while the Ceratopteris lateral meristem is a multicellular meristem (Geng et al. 2022). It is therefore perhaps no surprise that they do not share the same response.

> *12. ¨Ceratopteris sporophytes closely resemble responses of Arabidopsis sporophytes¨. This response was partially demonstrated 20 years ago by Hou etal (2004). Unfortunately, the description of the results shown in Figure 2 is ambiguous as an example the following paragraph: ¨This increased branching was pronounced after 12 days of growth in auxin supplemented medium (Fig. 2C,E), but an initial response was already visible after three days (Fig. S2A,B). Interestingly, the increased branching was not visible in the first appearing root of the young sporophytes, clearly showing that branching is root age/stage- dependent, and potentially explaining why this response is sometimes not visible¨. These results are poorly analyzed and described, one would expect that authors would describe and emphasize the novel findings compared to Hou et al 2004, since Hou and coworkers already showed the effect of diverse auxinic molecules over the fifth root of Ceratopteris with great detail. They already showed that the response of the root to exogenous auxin is stage-dependent. Indeed in the discussion section, the authors claim: ¨In summary, by studying the fern Ceratopteris richardii, a missing link in our understanding of auxin biology has been filled¨ this implies that their work for the first time fills the gap, when Hou et al did it for the sporophyte in 2004 and Withers metal, for the gametophyte in 2023. Instead of such a statement, one would expect that authors could discuss how their work coincides, or not, with previous studies, and integrate the findings of the three studies. Once their results are well described. Another major observation is the description of the analyses of exogenous auxin on Arabidopsis root hair development versus those of Ceratopteris in the roots of the sporophyte. What are the conclusions, similarities, or differences between rhizoids and root hairs?*

We thank the reviewer for bringing this important point to our attention. Our intention was not to disregard or downplay previous research but apparently we have to clarify this much better in the text. We have rewritten this paragraph extensively and made clearer references to the stated literature and the added value of our results. Those papers were already mentioned in the results section but we made it more pronounced. We did this both for the first gametophyte and second sporophyte section.

Additionally, we tried to put the Ceratopteris responses in a wider evo-devo perspective by also including *Marchantia* to a major and *Arabidopsis* to a minor extent in the result section. And as we mention in the introduction, by also taking both generations of Ceratopteris into account, we hope to achieve a wider developmental and evolutionary understanding of these auxin responses and their conservation among all land plants.

> *13. ¨Disrupting auxin transport can induce a sporophytic program¨ This could be a major finding of this study, however, it is not presented in a detailed manner to avoid a wrong conclusion. Authors claim that inhibiting the transport of auxin causes a major reprogramming of the gametophyte to a sporophyte and they sustain this by the observation of root formation after prolonged treatment with NPA. However to sustain that NPA induces the formation of ectopic roots from tissues of non-fertilized hermaphrodite gametophytes, a time-lapse of the process should be registered. How can authors discard that during the prolonged treatment the self-fertilization of the hermaphrodite gametophyte occurred and the root emergence is only accelerated? Authors show a single picture of putative root apical cell, how often this occurs in NPA treatment versus non-treated gametophytes? This never occurred in non-treated gametophytes cultured during the same, prolonged, time?. Are these claimed ¨transdifferentiation¨ events occurring also in the male gametophyte or only in the hermaphrodite? A time-lapse observation versus the control recorded and in a system in which during the prolonged culture could not cause self-fertilization could strengthen and make more robust this interesting observation.*

We thank the reviewer for sharing this concern. We added an additional supplemental figure (Fig. S5) to show a time series of development. We also added an extra description in the manuscript stating: ‘’Upon closer inspection, NPA treated plants show abnormal cellular morphologies close to archegonia, which mark the locations where later on root-like apical cells appear and roots grow out

© 2024. Published by The Company of Biologists under the terms of the Creative Commons Attribution License (https://creativecommons.org/licenses/by/4.0/).

(Fig. S5A). These roots do not originate from an egg cell or zygote for multiple reasons: (1) they originate a few cells away from the archegonia; (2) they produce vascular tissues which is distributed randomly in the gametophyte and not attached to a zygotic leaf (Fig. 3B) and (3) the roots develop much faster than zygotic roots (Fig. S5) (Aragón- Raygoza et al., 2020).". Additionally, on prolonged culturing under normal conditions of these ectopic roots on the gametophyte, no sporophytic leaf is formed, which would be the case if it is zygotic. This is logical as the gametophytes had no fertilization event (liquid water).

Regarding the non-treated gametophytes, there the cellular patterns are much more distinct and no active cell divisions take place around the archegonia, as also shown by Geng et al. (2022). However, the NPA-treated gametophytes are much less ordered and seem more random. Also, groups of smaller cells can be detected around archegonia (Fig. S5A, top left panel) which suggest an induction of division prior to RAC establishment which normally does not happen in non-treated gametophytes. Additionally, we did H2B quantification in NPA-treated gametophytes compared to mock treated gametophytes, and this showed no clear difference, suggesting the ploidy only changes in the root-initials.

Regarding the control cultured for such a long time. This induction of roots of gametophytes can be achieved within 20 days after plating the spores. Normal hermaphrodite gametophytes grown for 20 or even 40 days develop no such ectopic roots. If there are roots, they are always part of a young sporophyte and thus attached to a leaf. In literature, a similar phenomenon has been reported, when grown on sugar plates for long term (>40 days), apogamy can happen in Ceratopteris gametophytes but they develop haploid sporophytic leaves which we do not see. Also, they do not detect any roots in this apogamous leaves (Corde et al. (2007)).

This event never happened in male gametophytes as they stop developing after sexual specification.

> *14. Finally, the most novel data generated by this study is the comparative transcriptomic profiling, in response to exogenous auxin, among Marchantia, Ceratopteris, and Arabidopsis. These analyses lead to the identification of up-regulated genes among which, as expected, are genes related to auxin-synthesis, transport, and signaling. Unfortunately, the bulk transcriptomic analyses do not allow the identification of cell-type specific genes that supports the hypothesis of a conserved response to auxin at cell type level. My major concern is again the final interpretation of the data which is too vague, and example of this is the following paragraph: ¨The transcriptional nature of the response clearly depends on the ontogeny of the organism and vice-versa shapes its development due to their interdependency¨;¨... we find that auxin may control the alternation of generations.¨ The phosphor-proteomics results are also quite different among species, but authors do not explain clearly why. Did they compare phosphor-proteomics from Marchantia thalli versus that of Ceratopteris hermaphrodite gametophyte? Yes or not and why not? Did they compare Arabidopsis roots versus Ceratopteris roots phosphor-proteins, Yes or not and why not? If the answer is not, how can authors and readers could conclude some reasonable comparison or claim ontogeny conservation? Could they elaborate more in the discussion?*

We again thank the reviewer for sharing these concerns, we have tried to clarify the text and our conclusions. Regarding the identification of cell-type specific genes, that was not our aim and also beyond the scope of this manuscript. To our knowledge, there are no reports of combining scRNA-seq with hormone treatments outside of the well-established Arabidopsis root system. Also, our main conclusion is not that all the targets are conserved but rather the nature of the response (activation versus repression; high of low amplitude of DEG).

Regarding the phospo-proteomics, we specified the comparisons in the text and tried to better explain the differences between the species and we clearly stated which tissues we compared. We compared Marchantia thallus, Physcomitrium gametophores, Ceratopteris hermaphrodite prothallus, Ceratopteris whole sporophytes and Arabidopsis roots. For Ceratopteris, we wanted to compare whole plants, to make for a fair comparison to the prothallus we used whole sporophytes instead of only roots because otherwise there would be no photosynthetic tissues. We tried also on Arabidopsis whole seedlings, but that did not work well. In text we added this whole paragraph to specify and hopefully answer the reviewers concerns.

© 2024. Published by The Company of Biologists under the terms of the Creative Commons Attribution License (https://creativecommons.org/licenses/by/4.0/).

The big advantage of these phosphor-proteomics comparison is that one can finally isolate both sporophytic and gametophytic tissues in large amounts. We also believe that the changes we see in each species are therefore an underestimation of the full effect, and that the comparative analysis across different tissues and generations underestimate the degree of conservation. There is no good way to avoid this, given the vast differences in anatomy. For example, it is not possible to sample enough material from Arabidopsis gametophytes or bryophyte sporophytes to do such comparisons. We feel that there is insufficient basis to use these differences as a component in data analysis as suggested.

14. Figure 1 legend, should be specific when referring to Marchantia or Ceratopteris, non-expert readers will be confused when authors are describing cells from one or the other. Some images in in Figure 1A, D and E are so small that it is difficult to observe what the authors claim, besides growth reduction. What are red asterisks indicating in images in Figure 1 Panel F? There are several typos along the text, please revise again.

We thank the reviewer for these suggestions to improve clarity. We adjusted the legend of Figure 1, indicated the meaning of the red asterisk and revised to remove all typos.

Reviewer 3:

15. I would suggest the use 'phase' as opposed to 'ontogeny'. The gametophyte ontogeny of bryophytes, ferns, and angiosperms differ. Same for sporophyte ontogeny. But here they are comparing gametophyte phases or sporophyte phases the result of meiosis or syngamy respectively.

We thank the reviewer for this comment. We adjusted the phrasing where appropriate to developmental phase.

16. Although not inaccurate, I think it is clearer to use 'phase' instead of 'generation'. As both phases are needed to move from one generation to the next (unless apospory or apogamy occurs).

We thank the reviewer for this suggestion but we believe this would reduce the readability of the manuscript if we would change generation also to phase, similar to the previous comment. Also, the alternation of generations (gametophyte/sporophyte) is a widely accepted description in botany and therefore not using this vocabulary would make the manuscript less in line with previous literature.

17. Line 81 - change to 'Some lycophytes'. The selaginellales and isoetales are heterosporous and endosporic - that is the gametophytes develop inside the spore while sometimes still attached to the sporophyte. So the phases are not always independent. These gametophytes also lack chlorophyll. Some lycopodiales gametophytes are colorless and buried underground.

Removed the Lycophytes statement, as their development is indeed completely different.

18. Line 94 - there are many experiments done with auxin in fern species such as Wetmore & Pratt, 1949; Voeller 1960; Briggs & Steve, 1959; Steve & Briggs, 1960; Peterson and Cutter 1969a,b ; Reviewed in White 1971 Botanical Review 37: 509-540. these should be included as relevant.

We thank the reviewer for these suggestions, and included all the references.

19. Line 101 - how do responses in physcomitrium gametophytes compare?

We mentioned Physcomitrum reponses shortly in the introduction. However, we mainly considered *Marchantia* due to its laminal morphology (thallus), which has more similarities to a Ceratopteris

© 2024. Published by The Company of Biologists under the terms of the Creative Commons Attribution License (https://creativecommons.org/licenses/by/4.0/).

gametophyte than Physcomitrium chloro/caulonema and gametophores. However, we agree this was now missing to much so we added a line in the discussion to bring this point more to the attention.

20. Line 125 - typo change to 'importantly'

Adjuste
d

21. Lines 132-135 - auxin has an affect on the egg cell of Arabidopsis. How far did you follow ontogeny of the ceratopteris archegonium? It is not possible to see the egg cell in supplemental Fig S1b. Also, did fertilization occur? Were there any impacts on embryo ontogeny? Auxin has a strong impact on embryo patterning in angiosperms.`

We thank the reviewer for bringing up this interesting point. We considered this beyond the scope of this manuscript as we could not make a good comparison to bryophyte embryos and therefore would miss the point of an evo-devo comparison across land plants. Therefore this would be more appropriate for a different manuscript.

In Fig S1b, part of an egg cell is visible in the two right panels. These pictures are confocal slices of a complete Z-stack and in all of them egg cells are present. Additionally, egg cell precursors (central cell) and neck precursors are specified first (Sokolof & Remizowa, 2021) and only afterwards the archegonia jacket/wall is only specified later which is visible in Fig S1B. So in Figure S1B an endpoint of archegonium development is visible and no obvious defects are visible.

22. Lines 134–5 - are there any auxin studies in physcomitrium?

Yes, we mentioned some in the introduction and later in the discussion. We also added a note that we mainly compare to *Marchantia* due to the thallus growth habit.

23. Line 136 - what are the red stars in panel F of fig 1?

We specified in the legend, they refer to the lateral meristems.

24. Lines 173-4 - laminar morphology of a gametophyte is not similar to euphyllophyte leaves.

We clarified the sentence to indeed prevent this unintended interpretation.

25. Line 175 - the evolution of leaves is far from settled. It is thought that leaves evolved once, twice (in lycophytes and again euphyllophytes (ferns + seed plants), or up to 12 times (mostly in the ferns). All data needs to be considered, fossil, morphological, and molecular. This is debate is far from settled and this sentence should reflect that.

We thank Reviewer 3 for the opportunity to clarify that important point. We agree with the reviewer that the precise number of times leaves evolved independently in the different plant groups is unclear. To the best of our knowledge, however, the recent debate is mainly on the number of times leaves evolved independently in ferns (e.g., Boyce & Knoll, 2002; Boyce, 2005; Sanders et al., 2009; Tomescu, 2009; Boyce, 2010). By contrast, there seems to be general agreement that leaves evolved independently in ferns and flowering plants as we stated. We made adjustments to the text to make this more clear.

26. Line 186 - were there any auxin affects on timing of sporangia development or position of sporangia or morphology of sporangia?

This is a good suggestion, but the simple answer is we do not know, we did not check. We only studied young sporophytic leaves as their morphology and venation is less complex and better to image then the later (fertile) stages. This is because of two additional reasons. (1) Practically, growing Ceratopteris sporophytes until sporulation takes a relatively long time, and from leaf

© 2024. Published by The Company of Biologists under the terms of the Creative Commons Attribution License (https://creativecommons.org/licenses/by/4.0/).

initiation to emergence and spore development is again a relative long time. Therefore, treatments would have to be very long (>30 days) and we can not guarantee the stability of the chemicals. Additionally, plants treated with either NPA or NAA grow generally more slowly.
(2) To our knowledge, there is no clear literature on bryophyte spore production and the role of auxin in that process. And sporogenesis in flowering plants carpels is a very derived process compared to fern leaves. Therefore, the comparative added value of studying sporangia development would be limited as there is no clear link to either bryophytes or seed/flowering plants.

27. Line 207 - where did these sporophyte roots arise from? Randomly? Near the notch meristem? On the rhizoid side of the gametophyte?

The sporophytic roots arise close to the notch meristem, in between the archegonia as is visible in Figure 4C. We added this addition to the text and to Fig. S5A.

28. Line 322 - why not knock these down Ceratopteris to directly assess the effect on duplications in aux/Iaa or a-type arf families?

We thank the reviewer for this comment, and we have considered this indeed. The only problem is that many duplications occurred in Ceratopteris (4 A-class ARFs, 16 Aux/IAAs, Fig S6) which would needed higher-order mutants. And with just recently the publication of the first two CRISPR mutants in Ceratopteris (Xiang et al. (2024), Jiang et al (2024), making a higher order mutant is quite ambitious. Also, because *Marchantia* has a minimal auxin system (1 A- class ARF, 1 Aux/IAA, see Kato (2015), Kato (2020) and Flores-Sandoval (2015)), adding or removing parts NAP components makes for much easier to interpret experiments, which we did for Figure 5.

29. Lines 422-4 - see comment on leaf evolution above. Also auxin experiments on ferns in another comment above.

We added an extra sentence to clarify the evolutionary perspective and added the other fern experiments, although they are not comparable as they studied compound fronds/leaves.

30. Lines 455 - typo change to 'advancements'

Adjusted.

31. Lines 455-7 - knockdowns are possible in ceratopteris - see bui et al, youngstrom et al, withers et al and placket et al. Along with recent reports of crispr in ceratopteris.

We added these extra references and rephrased the sentence to emphasize that we would need higher order mutants and that becomes more feasible with recent contributions from the mentioned references.

Second decision letter

MS ID#: DEVELOP/2024/203026

MS TITLE: Analysis of auxin responses in the fern Ceratopteris richardii identifies developmental phase as a major determinant for response properties

AUTHORS: Sjoerd Woudenberg, Melissa Dipp Alvarez, Juriaan Rienstra, Victor Levitsky, Victoria Mironova, Enrico Scarpella, Andre Kuhn, and Dolf Weijers
ARTICLE TYPE: Research Article

I am happy to tell you that your manuscript has been accepted for publication in Development, pending our standard publication integrity checks.

© 2024. Published by The Company of Biologists under the terms of the Creative Commons Attribution License (https://creativecommons.org/licenses/by/4.0/).
